# Exploring the Interspecific Interactions and the Metabolome of the Soil Isolate Hylemonella gracilis

**DOI:** 10.1128/msystems.00574-22

**Published:** 2022-12-20

**Authors:** Olaf Tyc, Purva Kulkarni, Adam Ossowicki, Vittorio Tracanna, Marnix H. Medema, Peter van Baarlen, W. F. J. van IJcken, Koen J. F. Verhoeven, Paolina Garbeva

**Affiliations:** a Netherlands Institute of Ecology (NIOO-KNAW), Department of Microbial Ecology, Wageningen, Netherlands; b Goethe University, Department of Internal Medicine I, University Hospital Frankfurt, Frankfurt, Germany; c Translational Metabolic Laboratory, Radboud University Medical Center, Nijmegen, Netherlands; d Institute of Biology, Above–Belowground Interactions Group, Leiden University, Leiden, Netherlands; e Wageningen University, Department of Plant Sciences, Bioinformatics Group, Wageningen, Netherlands; f University Cologne, Institut für Pflanzenwissenschaften, Cologne, Germany; g Wageningen University, Department of Animal Sciences, Host-Microbe Interactomics, Wageningen, Netherlands; h Center for Biomics, Erasmus University Medical Center, Rotterdam, Netherlands; i Netherlands Institute of Ecology (NIOO-KNAW), Department of Terrestrial Ecology, Wageningen, Netherlands; j Department of Plant and Environmental Sciences, Faculty of Natural and Life Sciences, University of Copenhagen, Copenhagen, Denmark; UMR1136 INRA Université de Lorraine

**Keywords:** *Hylemonella* sp., interspecific interactions, transcriptome analysis, metabolome analysis, Gram-negative bacteria, metabolomics, transcriptomics, volatile organic compounds

## Abstract

Microbial community analysis of aquatic environments showed that an important component of its microbial diversity consists of bacteria with cell sizes of ~0.1 μm. Such small bacteria can show genomic reductions and metabolic dependencies with other bacteria. However, so far, no study has investigated if such bacteria exist in terrestrial environments like soil. Here, we isolated soil bacteria that passed through a 0.1-μm filter. The complete genome of one of the isolates was sequenced and the bacterium was identified as Hylemonella gracilis. A set of coculture assays with phylogenetically distant soil bacteria with different cell and genome sizes was performed. The coculture assays revealed that *H. gracilis* grows better when interacting with other soil bacteria like *Paenibacillus* sp. AD87 and Serratia plymuthica. Transcriptomics and metabolomics showed that *H. gracilis* was able to change gene expression, behavior, and biochemistry of the interacting bacteria without direct cell-cell contact. Our study indicates that in soil there are bacteria that can pass through a 0.1-μm filter. These bacteria may have been overlooked in previous research on soil microbial communities. Such small bacteria, exemplified here by *H. gracilis*, can induce transcriptional and metabolomic changes in other bacteria upon their interactions in soil. *In vitro*, the studied interspecific interactions allowed utilization of growth substrates that could not be utilized by monocultures, suggesting that biochemical interactions between substantially different sized soil bacteria may contribute to the symbiosis of soil bacterial communities.

**IMPORTANCE** Analysis of aquatic microbial communities revealed that parts of its diversity consist of bacteria with cell sizes of ~0.1 μm. Such bacteria can show genomic reductions and metabolic dependencies with other bacteria. So far, no study investigated if such bacteria exist in terrestrial environments such as soil. Here, we show that such bacteria also exist in soil. The isolated bacteria were identified as Hylemonella gracilis. Coculture assays with phylogenetically different soil bacteria revealed that *H. gracilis* grows better when cocultured with other soil bacteria. Transcriptomics and metabolomics showed that *H. gracilis* was able to change gene expression, behavior, and biochemistry of the interacting bacteria without direct contact. Our study revealed that bacteria are present in soil that can pass through 0.1-μm filters. Such bacteria may have been overlooked in previous research on soil microbial communities and may contribute to the symbiosis of soil bacterial communities.

## INTRODUCTION

Bacteria are ubiquitous living organisms with various cell shapes and sizes surrounding us in all environments (1, 2). Soil is the most complex habitat harboring the largest diversity and density of bacteria known to date (cell densities ranging from 10^7^ to 10^10^ cells/g of soil) (3–5). Soil bacteria are part of a community where they are in constant interaction with their own and other species (6–8). Bacteria produce and release a plethora of metabolites into their environment. In this way, they not only chemically modify their niche but also affect the behavior and the secondary metabolite production of nearby bacteria (9–11). Soil bacteria are known to produce a wide range of soluble and volatile secondary metabolites with different physicochemical and biological properties (7, 12–14). In contrast to soluble compounds, volatile organic compounds (VOCs) are rather small molecules (<300 Da) that can diffuse easily through air- and water-filled soil pores (15–17). These physicochemical properties make VOCs ideal metabolites for long-distance communication and interactions between soil microorganisms (18–21).

In aquatic environments, bacteria are naturally found at lower cell densities compared to soil (10^3^ to 10^6^ cells/mL) (22–24). Recent studies have shown that a significant component of aquatic microbial diversity consists of bacteria with small cell sizes of about ~0.1 μm (25–27). However, little is known about bacteria with such cell sizes in soil environments, for instance in water-filled soil pores. There are indications that ultra-small bacteria might exist in rice paddy soils as described by Jansen (28) as “dwarf cells” and in other soil environments (29).

One can assume that a small cell size can be an advantage in challenging environments like soil. However, the distribution of microorganisms in soil is influenced by its water and moisture content, and a low soil moisture content leads to lower connectivity between soil pores, and thus to a lower number of accessible microhabitats.

Small bacterial cell size is often linked to a small genome size caused by genome streamlining (30). Recent metagenomic studies suggest that genome streamlining is ubiquitous in bacteria (31, 32). In some cases, the primary metabolism of one organism can be directly built on the primary metabolism of another organism, known as syntrophic relationships (33, 34). The Black Queen Hypothesis states that genome-streamlined organisms have an evolutionary advantage because of the loss of genes whose function can be replaced by bacteria in the surrounding environment, effectively conserving energy (35). Since bacteria with fewer genes have less adaptive capacity compared to bacteria with more genes, many of them are expected to depend on specific environmental conditions or on the presence of other specific organisms (36) to produce metabolites that support their persistence.

Here, we aimed to explore if bacteria that can pass through 0.1-m filters are present in soil, and if such bacteria are cultivable. We further investigated their interaction with phylogenetically different bacteria commonly occurring in soil. The major research questions were if, and how interspecific interactions between bacteria that pass a 0.1-μm filter and other common soil bacteria that cannot pass 0.1-μm filters affected their fitness, behavior, gene expression, and the production of secondary metabolites.

## RESULTS

### Isolation and identification of bacteria that pass through 0.1-μm filter.

We isolated bacteria from a terrestrial soil sample that were able to pass through 0.22-μm and 0.1-μm pore size filters. After several days of incubation, only one type of bacterial colonies was observed on the inoculated plates. The grown colonies were identified as Hylemonella gracilis (Gram-negative; class, *Betaproteobacteria*; order, *Burkholderiales*) by 16S rRNA sequence analysis.

**FIG 1:**
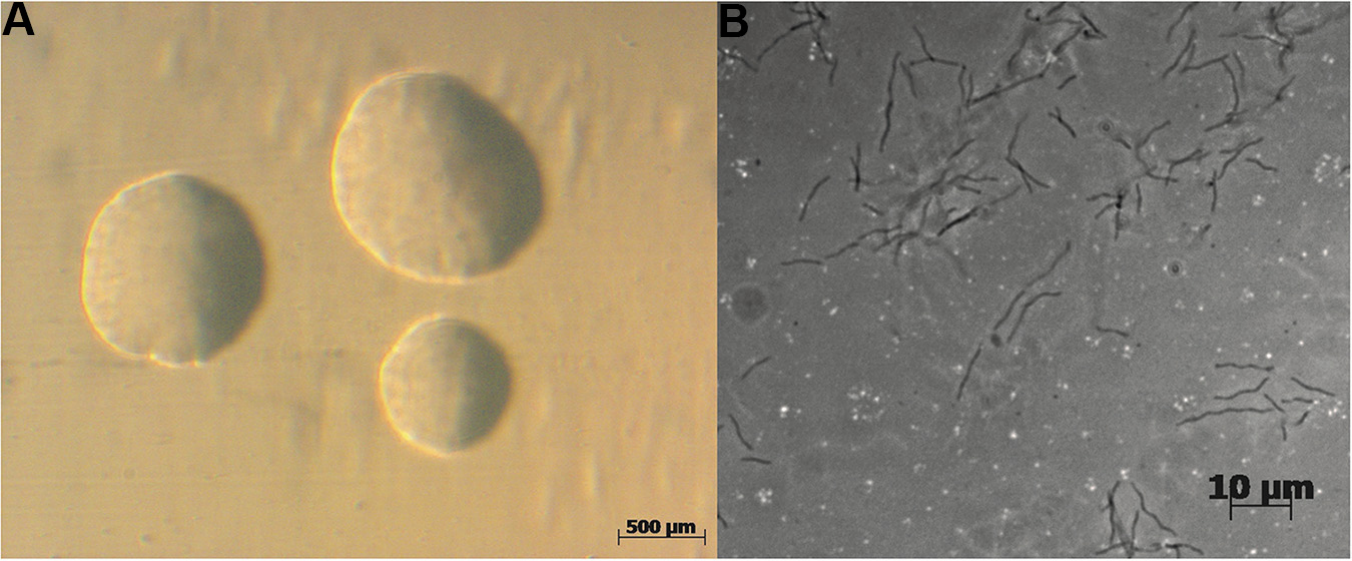
Morphology of Hylemonella gracilis (A) on 1/10th TSB agar plates captured at ×20 magnification and (B) single bacterial cells captured at ×400 magnification showing their very thin, long, and slender appearance in liquid media.

The colonies showed a round and colorless morphology when grown on 1/10th TSBA plates (Fig. 1A). Microscopically, the bacteria had a spiraled morphology with a length of approximately 6 to 12 μm, which is typical for *Hylemonella* species (Fig. 1B).

### *Hylemonella* grows better in interaction with other bacteria.

**FIG 2:**
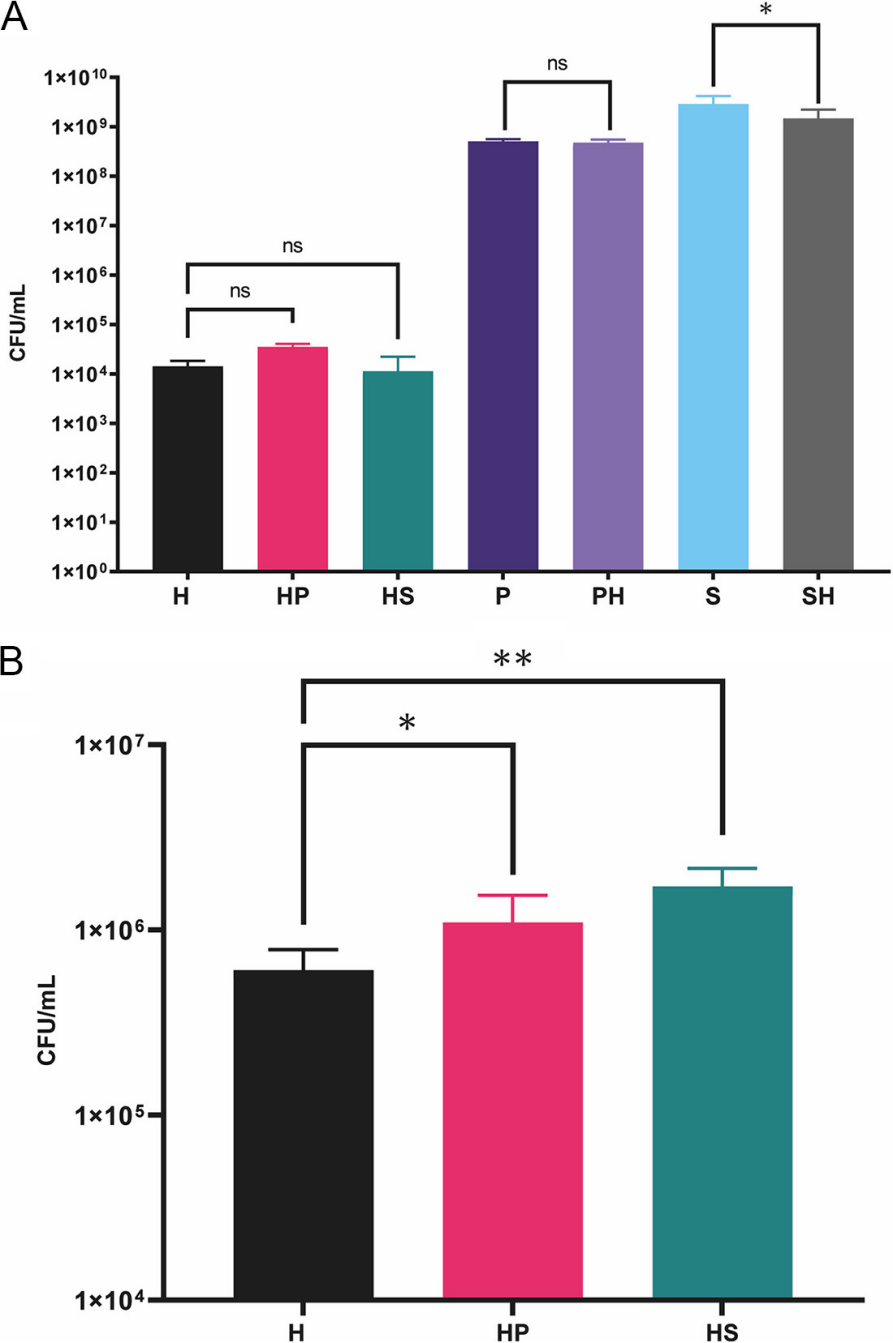
Growth of bacterial mono- and cocultures (A) on the plate-based experiment *, *P* < 0.05 (*P* = 0.037); and (B) during the cell-free-supernatant (CFS) experiment. H, *H. gracilis* monoculture; HP, *H. gracilis* coculture with *Paenibacillus* sp. AD87 (cell numbers for *H. gracilis*); HS, *H. gracilis* coculture with *S. plymuthica* PRI-2C (cell numbers for *H. gracilis*); P, *Paenibacillus* sp. AD87 monoculture; PH, *H. gracilis* coculture with *Paenibacillus* sp. AD87 (cell numbers for *Paenibacillus* sp. AD87); S, *S. plymuthica* PRI-2C monoculture; SH, *H. gracilis* coculture with *S. plymuthica* PRI-2C (cell numbers for *S. plymuthica* PRI-2C). Significant differences in CFU per milliliter (CFU/mL) between cocultures (treatment) and monocultures (controls) are indicated by asterisks (one-way ANOVA, *post hoc* Tukey’s test). *, *P* < 0.05 (*P* = 0.011) **, *P* < 0.01 (*P* = 0.000).

To test the hypothesis that small bacteria grow better in the presence of normal-sized bacteria, growth of *H. gracilis* was determined in coculture with two phylogenetically distantly related soil bacteria (*Paenibacillus* sp. AD87 and Serratia plymuthica PRI-2C) and compared to that of the monoculture. The bacterial CFU of *H. gracilis* (CFU/mL) obtained on 1/10th TSBA plates from monocultures and cocultures are summarized in Fig. 2. Cell counts of *Paenibacillus* sp. AD87 were 7.68 × 10^7^ CFU/mL in coculture with *H. gracilis* (Fig. 2A). During the interaction with *H. gracilis*, the growth of *S. plymuthica* was significantly negatively affected (*P* = 0.037) after 5 days of incubation by reaching 1.47 × 10^9^ CFU/mL compared to the monocultures (Fig. 2A).

The bacterial CFU obtained from *H. gracilis* grown in the presence of cell free supernatants (CFS) of *Paenibacillus* sp. AD87 and of *S. plymuthica* are summarized in Fig. 2B. *H. gracilis* growth was significantly increased (*P* = 0.011) when growing in the presence of CFS of *Paenibacillus* sp. AD87, resulting in higher *H. gracilis* cell counts compared to the monoculture by reaching 1.10 × 10^6^ CFU/mL. In the presence of CFS from *S. plymuthica* PRI-2C, *H. gracilis* reached the highest cell counts at 1.72 × 10^6^ CFU/mL (*P* = 0.000) after 5 days of incubation (Fig. 2B).

### Interspecific interaction between bacterial species allows use of additional substrates.

**FIG 3:**
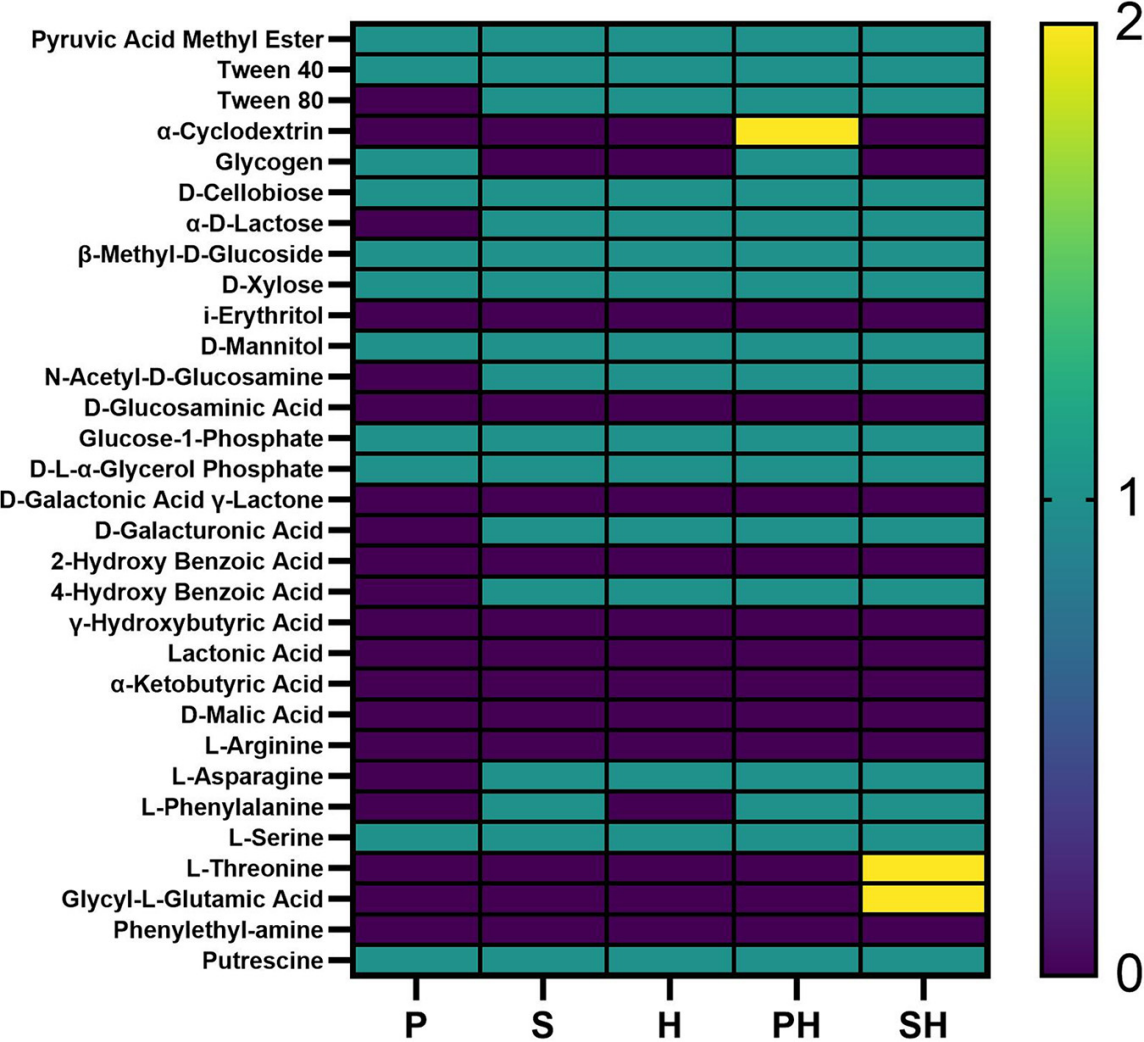
Results of the Biolog EcoPlate experiment. Bacteria were inoculated in monoculture or in pairwise combinations on the EcoPlate with 31 different carbon sources. Color code: turquoise indicates carbon source could be utilized in monoculture (1); yellow indicates carbon source could be utilized only in coculture (2); purple indicates carbon source could not be utilized (0). *Paenibacillus* sp. AD87 monoculture (P); *H. gracilis* coculture with *Paenibacillus* sp. AD87 (PH); *H. gracilis* monoculture (H); *S. plymuthica* PRI-2C monoculture (S), *H. gracilis* coculture with *S. plymuthica* PRI-2C (SH).

During physiological or catabolic profiling, the metabolism of 31 carbon sources during bacterial growth are measured in 96-wells plates. The catabolic profiling assays revealed that *Paenibacillus* sp. AD87was able to utilize 11 out of the 31 carbon sources in monoculture, while *S. plymuthica* PRI-2C and *H. gracilis* were able to utilize 17 and 16 carbon sources, respectively. Interestingly, three compounds could be utilized only during the cocultivation of *H. gracilis* with one of the other species, but not by any of the monocultures. Specifically, α-cyclodextrin was utilized only during the cocultivation of *H. gracilis* with *Paenibacillus* sp. AD87, while l-threonine and glycyl-l-glutamic acid were utilized only during the cocultivation of *S. plymuthica* PRI-2C and *H. gracilis* (Fig. 3).

### Genomic features of *H. gracilis*, *S. plymuthica* PRI-2C, and *Paenibacillus* sp. AD87.

Sequencing of the complete genome of *H. gracilis* resulted in a genome size of 3.82 Mbp with 3,648 coding sequences (CDS). As expected, the genome analysis revealed that the genome of *H. gracilis* is smaller and contains fewer genes compared to *S. plymuthica* PRI-2C (5.4 Mbp) and *Paenibacillus* sp. AD87 (7.0 Mbp). The genome features of all three bacteria are summarized in Table 1.

**TABLE 1:** Genome assembly statistics and outcome of *in silico* analysis of secondary metabolite gene clusters of *H. gracilis*, *S. plymuthica* PRI-2C and *Paenibacillus* sp. AD87

Feature/organism	*H. gracilis*	*S. plymuthica* PRI-2C	*Paenibacillus* sp. AD87
Contigs	1	1	30
Bases	3,822,245	5,474,685	7,086,713
No. of chromosomes	1	1	1
Size chromosome 1	3,822,245	5,464,425	7,086,713
CDS	3,648	4,929	6,216
GC content (%)	65.1	55.7	46.2
No. of RNAs	53	109	146
Genes	3,625	5,284	6,375
*In silico* detected secondary metabolite clusters (antiSMASH)	3	9	10
Total genome size (bases)	3,822,245	5,474,685	7,086,713

### *In silico* analysis of gene clusters encoding secondary metabolites.

**FIG 4:**
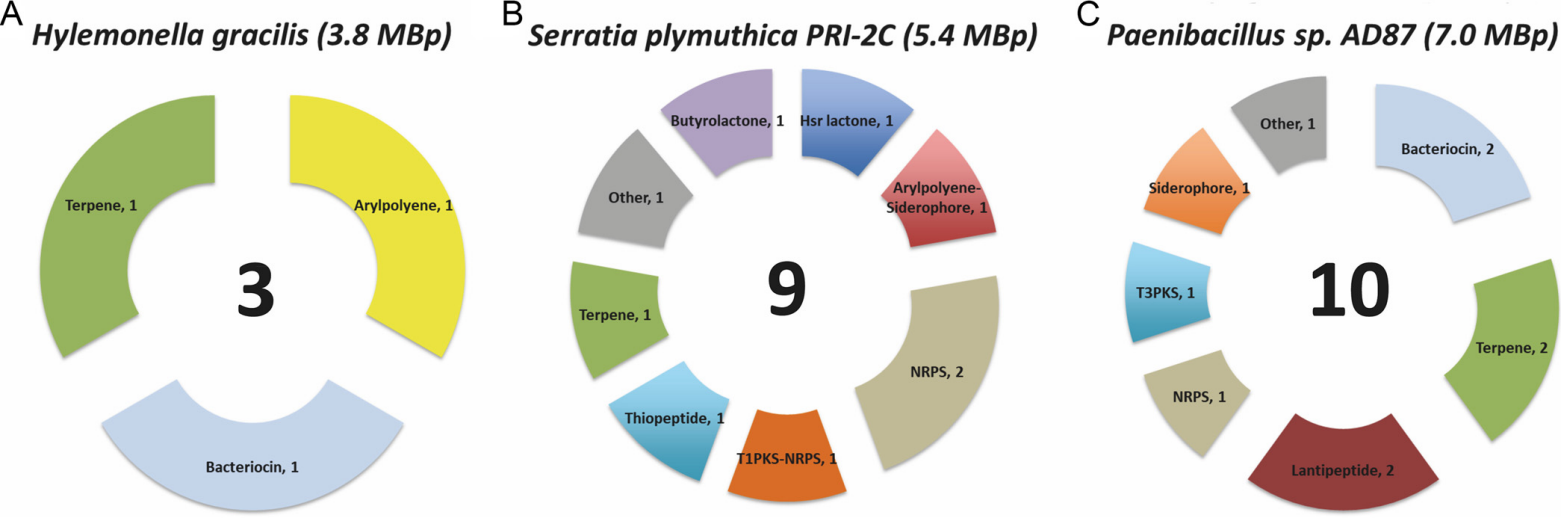
*In silico* comparison of biosynthetic gene clusters (BGCs) predicted by antiSMASH in the genomes of three soil bacteria. From left to right: (A) *H. gracilis* with a genome size of 3.8 MBp, *n* = 3 gene clusters for secondary metabolites; (B) *S. plymuthica* PRI-2C with a genome size of 5.4 MBp, *n* = 9 gene clusters for secondary metabolites; and (C) *Paenibacillus* sp. AD87 with a genome size of 7.0 MBp, *n* = 10 gene clusters for secondary metabolites.

The *in silico* tool *antiSMASH* allows the rapid genome-wide identification, annotation, and analysis of secondary metabolite biosynthesis gene clusters in bacterial and fungal genomes (37). *In silico* analysis of *H. gracilis* revealed that *H. gracilis* possesses relatively few gene clusters related to secondary metabolism. A total of three gene clusters for *H. gracilis* were detected, of which one belonged to the class of bacteriocins, one to the class of terpenes, and one to aryl polyenes, the latter being a homolog to the aryl polyene gene cluster from Xenorhabdus doucetiae (GenBank accession no. NZ_FO704550.1) (Fig. 4A).

For *S. plymuthica* PRI-2C, nine gene clusters were found, of which two gene clusters were annotated to encode the production of nonribosomal peptides (NRPs), one of homoserine lactones, one of aryl polyenes and/or non-NRP siderophores, one of hybrid polyketide-NRP metabolites, one of thiopeptides, one of butyrolactones, one of terpenes, and one of others (Fig. 4B).

For *Paenibacillus* sp. AD87 the *in silico* analysis revealed a total of 10 gene clusters coding for secondary metabolites. From which two gene clusters encode pathways for producing terpenes, one for bacteriocins, one for lasso peptides, two for lanthipeptides, one for NRPs, one for others, one for polyketides (type III enzyme mechanism) and one gene cluster for non-NRP Siderophores (Fig. 4C).

### Pathway analysis in *H. gracilis* compared to *S. plymuthica* PRI-2C and *Paenibacillus* sp. AD87.

**FIG 5:**
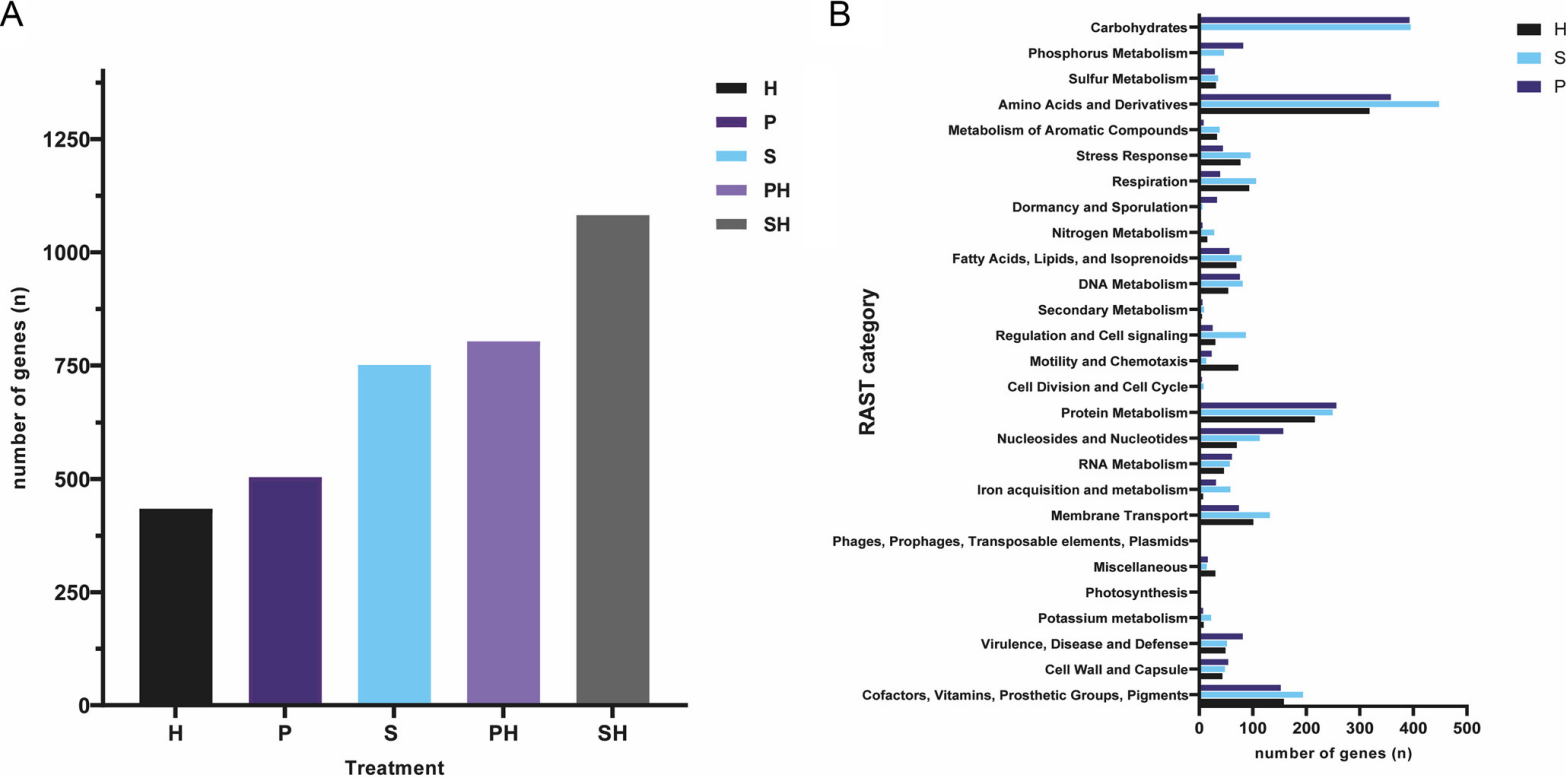
Gene content comparison. (A) Box plot showing the number of all expressed genes (*n*) for the monocultures of *H. gracilis* (H), *Paenibacillus* sp. AD87 (P), and *S. plymuthica* PRI-2C (S), and during the interactions of *H. gracilis* with *Paenibacillus* sp. AD87 (PH) and of *H. gracilis* with *S. plymuthica* PRI-2C (SH) determined by RAST. (B) Box plot showing number of expressed genes present in each RAST subsystem category for each of the monocultures.

For annotation and pathway analysis Rapid Annotation using Subsystem Technology (RAST) and OrthoFinder were used. The RAST comparison of *Paenibacillus* sp. AD87 and *H. gracilis* revealed 504 unique enzymes (according to their Enzyme Commission (EC) numbers.) exclusive for *Paenibacillus* sp. AD87, while 434 were present only in *H. gracilis* and 532 EC numbers were shared by both genomes (Fig. 5A). The RAST comparison of *S. plymuthica* PRI-2C and *H. gracilis* revealed that 751 enzymes were present only in *S. plymuthica* PRI-2C, and 260 were present only in *H. gracilis*. A total of 727 EC numbers participating in diverse metabolic pathways were found in both genomes (Fig. 5B).

The missing genes and pathways found by OrthoFinder and EggNOG were annotated with Gene Ontology (GO) terms. The analysis revealed that five genes related to metabolic pathways were absent in *H. gracilis*. Those missing genes were annotated with the following molecular function ontology terms: GO:0008473 (ornithine cyclodeaminase activity), GO:0008696 (4-amino-4-deoxychorismatelyase activity), GO:0003920 (GMP reductase activity), GO:0004035 (alkaline phosphatase activity), and GO:0008442 (3-hydroxyisobutyrate dehydrogenase). We verified if the absence of these molecular functions would render specific pathways obsolete or unavailable in *H. gracilis*. However, alternative pathway routes are present for these genes encoding certain molecular functions according to KEGG database annotations. The pathway analysis by RAST did not reveal the absence of essential genes in *H. gracilis*. Still, the comparison of the number (*n*) of genes present in each bacteria revealed major differences in several pathways, specifically in the categories “Carbohydrates metabolism” and “Phosphorus metabolism” (Fig. 5B). Interestingly, *H. gracilis* possesses no genes for those categories according to RAST, whereas *Paenibacillus* sp. AD87 possesses 393 and 82 genes, and *S. plymuthica* PRI-2C 395 and 46 genes, respectively. A major difference in the absolute number of genes in a category is also observed for amino acids and derivatives, for which *H. gracilis* possesses 318 genes, *Paenibacillus* sp. AD87 possesses 358, and *S. plymuthica* PRI-2C possesses 448 genes.

### Effect of interspecific interactions on gene expression.

The transcriptome analysis of monocultures and cocultures revealed a total of 277 significant differentially expressed genes; where 100 genes were downregulated and 177 genes were upregulated between the different treatments (Table 2).

**TABLE 2:** Overview of the transcriptome analysis, number (*n*) of significantly differentially expressed genes of *H. gracilis* responding to *S. plymuthica* PRI-2C or to *Paenibacillus* sp. AD87, Serratia plymuthica PRI-2C responding to *H. gracilis* and *Paenibacillus* sp. AD87 responding to *H. gracilis* at day 5 and day 10

Organism	Interacting organism	Time point (d)	Significantly differentially expressed genes		Total (*n*)
			Upregulated	Downregulated	
Serratia plymuthica PRI-2C	Hylemonella gracilis	5	25	36	61
		10	10	0	10
*Paenibacillus* sp. AD87		5	8	0	8
		10	0	0	0
Hylemonella gracilis	Serratia plymuthica PRI-2C	5	1	0	1
		10	129	53	182
	*Paenibacillus* sp. AD87	5	0	0	0
		10	4	11	15
Total (*n*)			177	100	277

**FIG 6:**
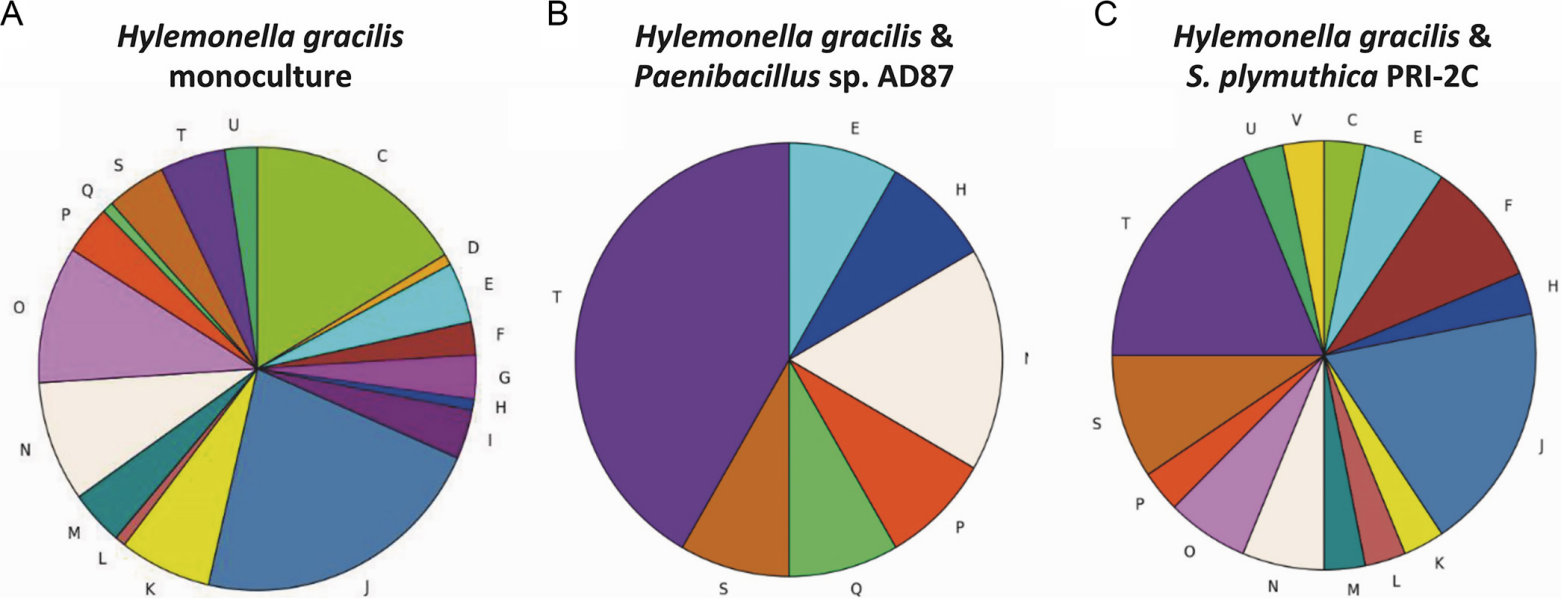
Pie charts representing upregulated genes identified by differential gene expression analysis and Clusters of Orthologous Genes (COG) annotation. (A) Hylemonella gracilis monoculture gene expression level; (B) *H. gracilis* in coculture with *Paenibacillus* sp. AD87; (C) *H. gracilis* coculture with *S. plymuthica* PRI-2C. In the coculture of *H. gracilis* with *Paenibacillus* sp. AD87, genes related to signal transduction (T) were the category with the most differentially expressed genes. In the coculture of *H. gracilis* with *S. plymuthica* PRI-2C, genes related to signal transduction (T), translation, ribosome structure and biogenesis (J) were the most prevalent differentially expressed gene categories. COG C, energy production and conversion; D, cell cycle control, cell division, and chromosome partitioning; E, amino acid transport and metabolism; F, nucleotide transport and metabolism; G, carbohydrate transport and metabolism; H, coenzyme transport and metabolism; I, lipid transport and metabolism; J, translation, ribosomal structure, and biogenesis; K, transcription; L, replication, recombination, and repair; M, cell wall/membrane/envelope biogenesis; N, cell motility; NA, not assigned; O, posttranslational modification, protein turnover, and chaperones; P, inorganic ion transport and metabolism; Q, secondary metabolites biosynthesis, transport, and catabolism; R, general function prediction only; S, function unknown; T, signal transduction mechanisms; U, intracellular trafficking, secretion, and vesicular transport; V, defense mechanisms.

**(i) Effect of interspecific interactions on gene expression in *Paenibacillus* sp. AD87 and *H. gracilis*.** Genes related signal transduction (T) was the category with the most differentially expressed genes during the cocultivation of *H. gracilis* with *Paenibacillus* sp. AD87 compared to the monoculture of *H. gracilis* (Fig. 6A and B, Table S6 and S7).

In *Paenibacillus* sp. AD87 histidine biosynthesis and dephosphorylation, genes were upregulated, while cellular-growth-related genes were downregulated (see Table S1 in the supplemental material) at day 10 of the interaction with *H. gracilis* (Fig. 6B). For the interaction of *H. gracilis* with *Paenibacillus* sp. AD87 15, significant differentially expressed genes were found (0 at day five and 15 at day 10). At day five, genes related to sulfur assimilation, chemotaxis, and response to (chemical/external) stimuli were upregulated in *H. gracilis* in the presence of *Paenibacillus* sp. AD87.

10.1128/msystems.00574-22.3 TABLE S1 Significantly differentially expressed genes of *Paenibacillus* sp. AD87, responding to *H. gracilis* at day 10. Download Table S1, PDF file, 0.08 MB. Copyright © 2022 Tyc et al. 2022 Tyc et al. https://creativecommons.org/licenses/by/4.0/ This content is distributed under the terms of the Creative Commons Attribution 4.0 International license.

**(ii) Effect of interspecific interactions on gene expression *S. plymuthica* PRI-2C and *H. gracilis*.** During the interaction of *S. plymuthica* PRI-2C with *H. gracilis*, 61 genes were significantly differentially expressed at day five and 10 at day 10. At day five, iron-sulfur cluster-assembly-related genes, a sulfur transferase, and a transaminase were upregulated, while genes related to inorganic diphosphatase activity, exonuclease activity, and DNA repair were downregulated. At day 10, genes related to sulfur transmembrane transport, sulfur compound catabolism, and cysteine biosynthesis were upregulated, and genes related to sulfur compound metabolism and translation were downregulated. (Table S2 and S3). For *S. plymuthica* PRI-2C, genes related to signal transduction and translation, ribosome structure, and biogenesis were the most differentially expressed gene categories (Fig. 6C). For *H. gracilis* in interaction with *S. plymuthica* PRI-2C, 182 differentially expressed genes were identified at day 10 and only one at day five. At day five, genes related to the ribosome/ribonucleoproteins, organelle organization/assembly, and iron-sulfur cluster assembly were upregulated, and genes related to the innate immune response (toll-like receptor signaling) were downregulated (Table S4 and S5). At day 10, genes related to signal transduction and chemotaxis were upregulated in *H. gracilis*. For *H. gracilis*, the most upregulated genes were linked to chemotaxis pathway and iron scavenging, suggesting activity in competition (Fig. 6A).

10.1128/msystems.00574-22.4 TABLE S2 Significantly differentially expressed genes of Serratia plymuthica PRI-2C responding to *H. gracilis* at day 5. Download Table S2, PDF file, 0.08 MB. Copyright © 2022 Tyc et al. 2022 Tyc et al. https://creativecommons.org/licenses/by/4.0/ This content is distributed under the terms of the Creative Commons Attribution 4.0 International license.

10.1128/msystems.00574-22.5 TABLE S3 Significantly differentially expressed genes of Serratia plymuthica PRI-2C responding to *H. gracilis* at day 10. Download Table S3, PDF file, 0.1 MB. Copyright © 2022 Tyc et al. 2022 Tyc et al. https://creativecommons.org/licenses/by/4.0/ This content is distributed under the terms of the Creative Commons Attribution 4.0 International license.

10.1128/msystems.00574-22.6 TABLE S4 Significantly differentially expressed genes of *H. gracilis* responding to Serratia plymuthica PRI-2C at day 5. Download Table S4, PDF file, 0.09 MB. Copyright © 2022 Tyc et al. 2022 Tyc et al. https://creativecommons.org/licenses/by/4.0/ This content is distributed under the terms of the Creative Commons Attribution 4.0 International license.

10.1128/msystems.00574-22.7 TABLE S5 Significantly up- or downregulated genes of *H. gracilis* responding to Serratia plymuthica PRI-2C at day 10. Download Table S5, PDF file, 0.08 MB. Copyright © 2022 Tyc et al. 2022 Tyc et al. https://creativecommons.org/licenses/by/4.0/ This content is distributed under the terms of the Creative Commons Attribution 4.0 International license.

### Metabolomic analysis of volatile compounds.

**FIG 7:**
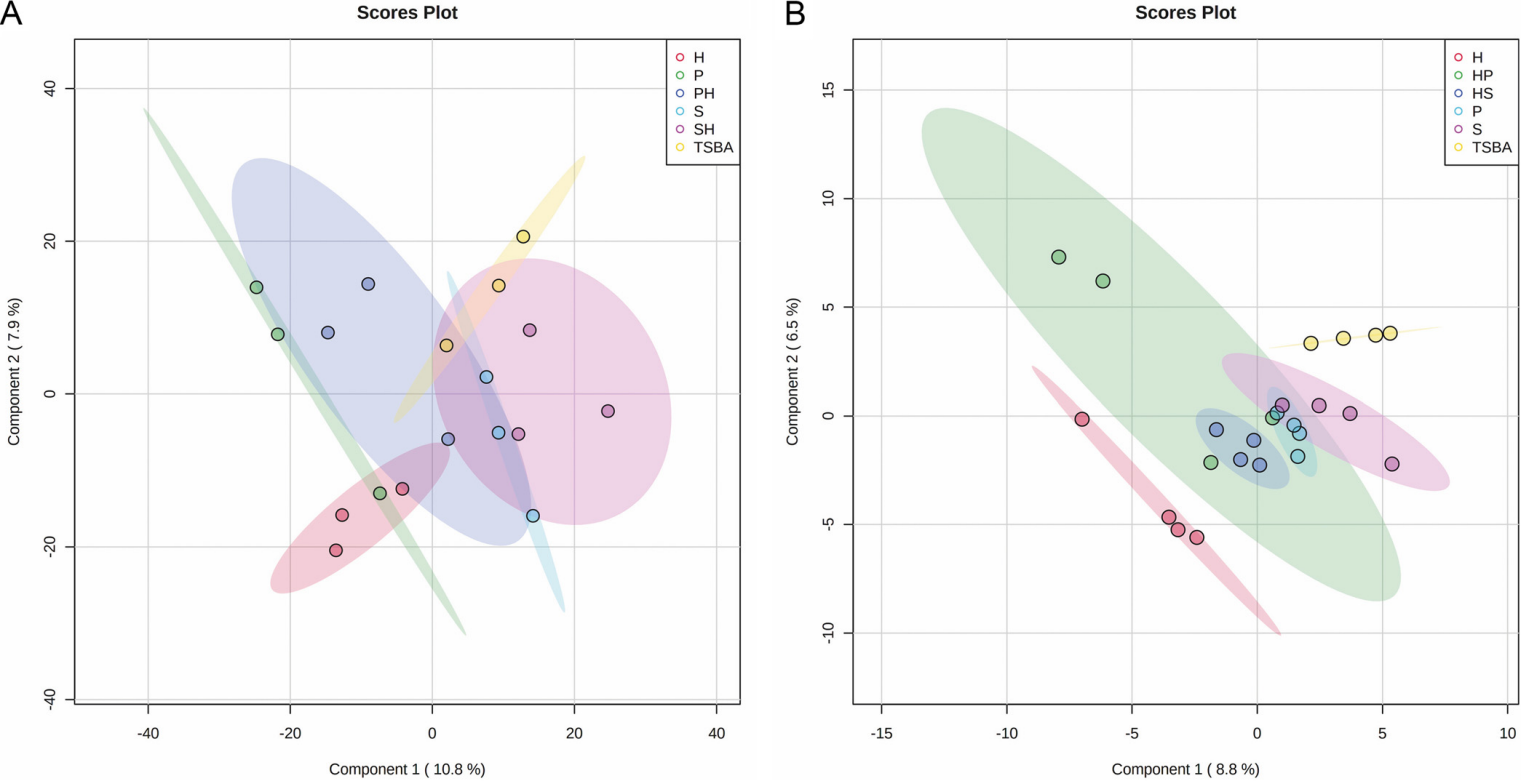
PLS-DA plots of the metabolomics data. (A) PLS-DA 2D plots of volatiles emitted by monocultures and pairwise combinations of *H. gracilis*, *Paenibacillus* sp. AD87, and S. plymuthica after 10 days of inoculation, time point (*t* = 10 days). (B) PLS-DA 2D plots of DART-MS data of monocultures and mixtures of *H. gracilis*, *Paenibacillus* sp. AD87, and *S. plymuthica* PRI-2C after 10 days of inoculation, time point (*t* = 10 days).

The volatile blend composition of the monocultures differed from that of the cocultures. Clear separations between the controls, monocultures and cocultures were obtained in Partial least squares discriminant analysis (PLS-DA) score plots (Fig. 7A). The analysis revealed a total of 25 volatile organic compounds produced by mono- and cocultured bacteria that were not detected in the noninoculated controls (Table 3). Of these, 17 were identified and categorized in six chemical classes (alkenes, benzenoids, sulfides, thiocyanates, terpenes, furans). The remaining eight compounds could not be assigned with certainty to a known compound. The most abundant volatile organic compounds were sulfur-containing compounds such as dimethyl disulfide (C_2_H_6_S_2_) and dimethyl trisulfide (C_2_H_6_S_3_). These two sulfur compounds were produced by all three bacteria. Interestingly an unknown compound with a retention time (RT) of 26.4 min produced by the monocultures of *H. gracilis* was not detected in the interactions with *S. plymuthica* PRI-2C (Table 3). Two other unknown compounds with an RT of 4.15 min and 24.34 min produced by the monocultures of *Paenibacillus* sp. AD87 were not detected in the cocultivation with *H. gracilis* (Table 3).

**TABLE 3:** Tentatively identified volatile organic compounds (VOCs) produced by *H. gracilis,* Serratia plymuthica PRI-2C, and *Paenibacillus* sp. AD87 strains in mono- and cocultures

Compound no.	Compound name	RT (m:s)^*a*^	ELRI^*b*^	*P* value^*c*^	Chemical class	Detected in bacterial culture^*d*^				
						H	S	P	SH	PH
1	2-Methylfuran	2.18	738	0.041	Furan	X		X	X	X
2	2-Methylpropanoic acid	3.01	755	0.014	Alkenes	X	X	X	X	X
3	Pentanal + heptane mixture	3.21	760	0.008	Alkenes	X			X	X
4	Methyl thiocyanate	3.44	764	0.020	Thioesters	X	X	X	X	X
5	1-Pentanol	3.95	772	0.012	Alkenes		X	X	X	X
6	Dimethyl disulfide	4.01	775	0.012	Sulfides	X	X	X	X	X
7	Unknown compound 1	4.15	778	0.003			X	X	X	
8	Toluene	4.44	784	0.014	Benzenoids	X	X	X	X	X
9	Methyl isovalerate	4.76	789	0.018	Terpenes		X	X	X	X
10	Cyclohexane	8.07	852	0.031	Alkenes		X	X	X	X
11	Dimethyl trisulfide	11.35	914	0.013	Sulfides	X	X	X	X	X
12	Benzonitrilie	12.06	928	0.037	Alkenes	X	X	X	X	X
13	2-Ethyl-4-methylpentan-1-ol	17.26	1,026	0.015	Alkenes		X	X	X	X
14	2,5-bis(1-methylethyl)-pyrazine	20.56	1,090	0.031	Pyrazines			X		X
15	Undecane	21.31	1,100	0.014	Alkenes	X		X	X	X
16	Unknown compound 2	24.34	1,140	0.013				X		
17	Unknown compound 3	25.92	1,160	0.011		X	X	X	X	X
19	Unknown compound 4	26.40	1,165	0.018		X		X	X	X
20	Unknown compound 5	26.90	1,170	0.003		X		X		X
21	Alpha-terpineol	27.34	1,178	0.016	Terpenes	X	X	X	X	X
22	Undecane, 2,6-dimethyl	28.27	1,190	0.004	Benzenoids	X	X	X	X	X
23	Gamma-terpineol	28.42	1,192	0.006	Terpenes	X			X	
24	Terpene-like compound 1	29.32	1,202	0.012	Terpenes	X			X	
25	Terpene-like compound 2	31.49	1,231	0.009	Terpenes	X	X	X	X	X
No. of detected compounds (*n*)						16	15	20	20	20

a RT, retention time in minutes and seconds. The RT value stated is the average of three technical replicates.

b ELRI, experimental linear retention index value. The value stated is the average of three replicates.

c *P* value, statistical significance (peak area and peak intensity).

d H, *H. gracilis* monoculture; SM, Serratia plymuthica PRI-2C monoculture; PM, *Paenibacillus* sp. AD87 monoculture; PH, *Paenibacillus* sp. AD87 and *H. gracilis* coculture; SH, Serratia plymuthica PRI-2C and *H. gracilis* coculture.

### Direct analysis in real-time mass spectrometry (DART-MS) based metabolomics.

Metabolomics analysis based on direct analysis in real-time mass spectrometry (DART-MS) revealed separations between the controls, monocultures, and cocultures as presented in PLS-DA score plots (Fig. 7B). The metabolomic composition of the monocultures differed from that of the cocultures (Fig. 7B). Statistical analysis (one-way ANOVA and *post hoc* Tukey’s HSD test) revealed 617 significant mass features present on day five and day 10 of which 48 could be tentatively assigned to specific compounds. Most of the significant peaks were found in the cocultures of *H. gracilis* with *Paenibacillus* sp. AD87. The significant mass features and the corresponding tentative metabolites can be found in Table 4.

**TABLE 4:** Detected and tentatively identified metabolites using DART-MS

Compound No.	*m/z*	Retention time (m:s)	Compound	*P* value	FDR value^*a*^
1	165.1384	0.20	Actinidine	0.000	0.000
2	223.0961	0.30	Apiole	0.007	0.037
3	249.148	0.40	1,2-Dihydrosantonin	0.007	0.037
4	137.1072	0.60	N,N-Dimethyl-1,4-phenylenediamine	0.006	0.037
5	110.0602	0.60	N-Vinyl-2-pyrrolidone	0.007	0.037
6	212.2005	0.70	Oxidized Latia luciferin	0.007	0.037
7	161.0806	0.70	Pimelate	0.000	0.000
8	132.1018	0.80	2-Hydroxycyclohexan-1-one	0.000	0.000
9	186.1486	0.80	6-Oxocineole	0.000	0.000
10	172.1329	0.80	Boschnialactone	0.006	0.037
11	227.0133	0.90	Bismuth	0.000	0.000
12	102.0916	0.90	Cyclopentanone	0.000	0.000
13	118.0863	1.00	5-Valerolactone	0.000	0.000
14	178.1337	1.10	Tryptamine	0.006	0.037
15	152.1432	1.20	p-Cymene	0.011	0.042
16	87.0087	1.30	Pyruvate	0.000	0.000
17	197.0222	1.50	5-methyl-3-isoxazolyl sulfate	0.009	0.037
18	175.0247	1.50	Ascorbate	0.006	0.037
19	121.0294	1.50	Benzoate	0.007	0.037
20	179.0559	1.50	D-Glucose	0.014	0.048
21	255.2328	1.50	Hexadecanoic acid	0.007	0.037
22	100.0759	1.50	Pentanamide	0.000	0.000
23	304.2477	1.60	2,3-Dihydroxycyclopentaneundecanoic acid	0.000	0.000
24	217.1795	1.70	12-Hydroxydodecanoic acid	0.000	0.000
25	123.0553	1.80	Nicotinamide	0.006	0.037
26	166.086	1.80	Trans-cinnamate	0.006	0.037
27	131.0713	1.90	R-2-Hydroxyisocaproate	0.000	0.000
28	129.0557	2.00	4-Methyl-2-oxopentanoate	0.006	0.037
29	253.2171	2.00	9Z-Hexadecenoic acid	0.006	0.037
30	164.0715	2.00	Coumarin	0.000	0.000
31	171.139	2.00	Decanoic acid	0.014	0.047
32	130.0873	2.30	L-Leucine	0.000	0.001
33	165.0191	2.40	Phthalate	0.000	0.000
34	117.0557	2.50	5-Hydroxypentanoate	0.000	0.000
35	101.0607	2.50	Pentanoate	0.000	0.000
36	134.0471	2.60	Adenine	0.000	0.000
37	241.2172	2.60	Pentadecanoic acid	0.000	0.000
38	128.0353	2.70	4-Oxoproline	0.000	0.005
39	130.0873	2.70	Cyclohexane-1,3-dione	0.000	0.000
40	227.2015	2.70	Tetradecanoic acid	0.007	0.037
41	116.9285	2.70	Unknown	0.000	0.000
42	187.1338	3.30	10-Hydroxydecanoic acid	0.000	0.000
43	243.1962	3.30	2S-Hydroxytetradecanoic acid	0.000	0.000
44	87.0329	3.40	Hydroxylamine hydrochloride	0.000	0.000
45	89.0243	3.50	Glycerone	0.000	0.000
46	116.0716	3.50	L-valine	0.006	0.037
47	224.999	3.60	Aminopyrrolnitrin	0.000	0.000
48	254.9731	3.60	Pyrrolnitrin	0.000	0.000

a FDR- value (false discovery rate) based on one-way ANOVA, followed by post hoc Tukey's test.

### Mass spectrometry imaging metabolomics.

**FIG 8:**
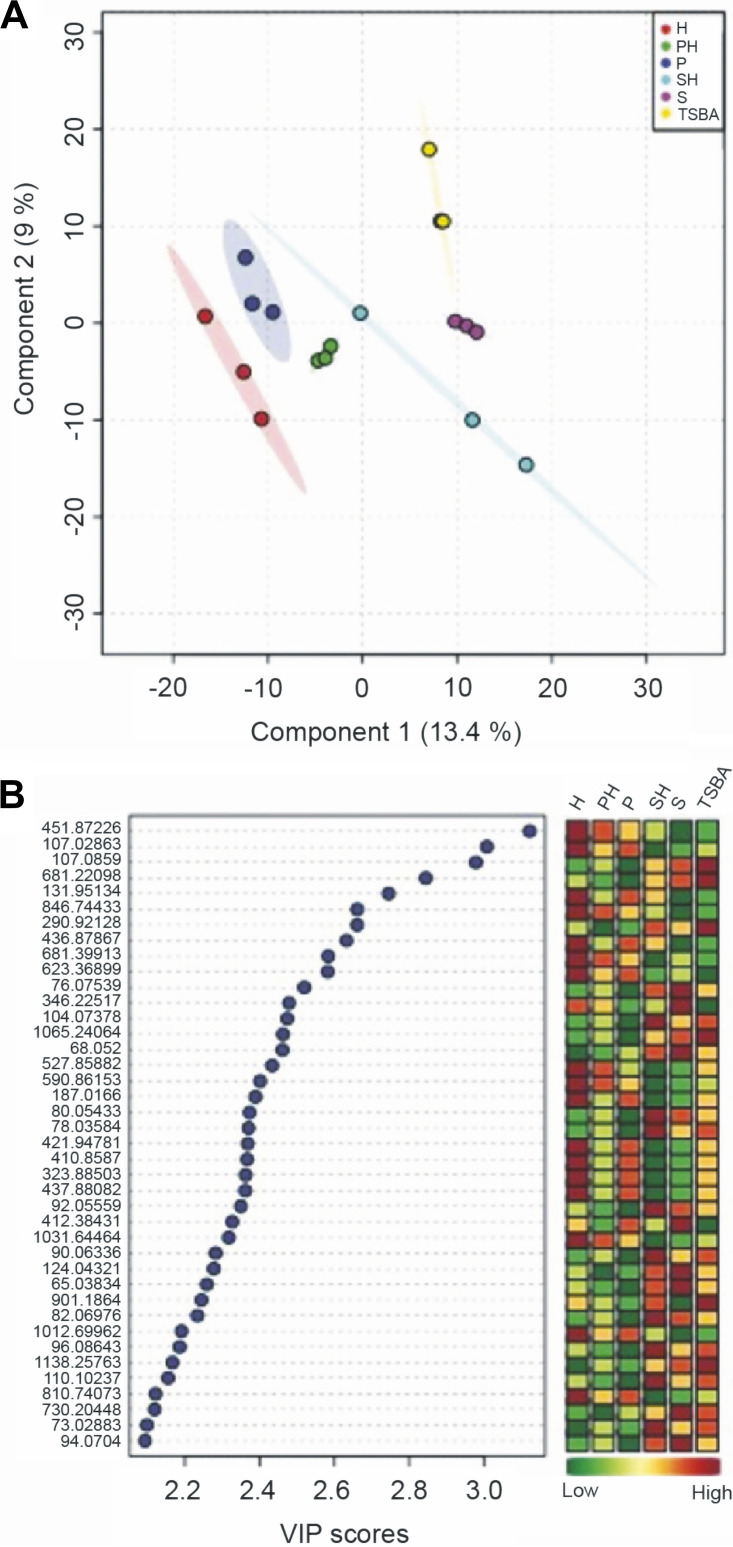
PLS-DA plots of the first 40 significant mass features observed in LAESI-MSI data. (A) PLS-DA score plot for *H. gracilis* monoculture (H), *Paenibacillus* sp. AD87 monoculture (P), *Paenibacillus* sp. AD87 with *H. gracilis* coculture (PH), *S. plymuthica* PRI-2C monoculture (S), *S. plymuthica* PRI-2C with *H. gracilis* coculture (SH), and TSBA control (TSBA). (B) Top 40 statistically significant features identified by PLS-DA based on variable importance in projection (VIP) score. The colored boxes on the right indicate the relative concentrations of the corresponding metabolite in each group under study.

**FIG 9:**
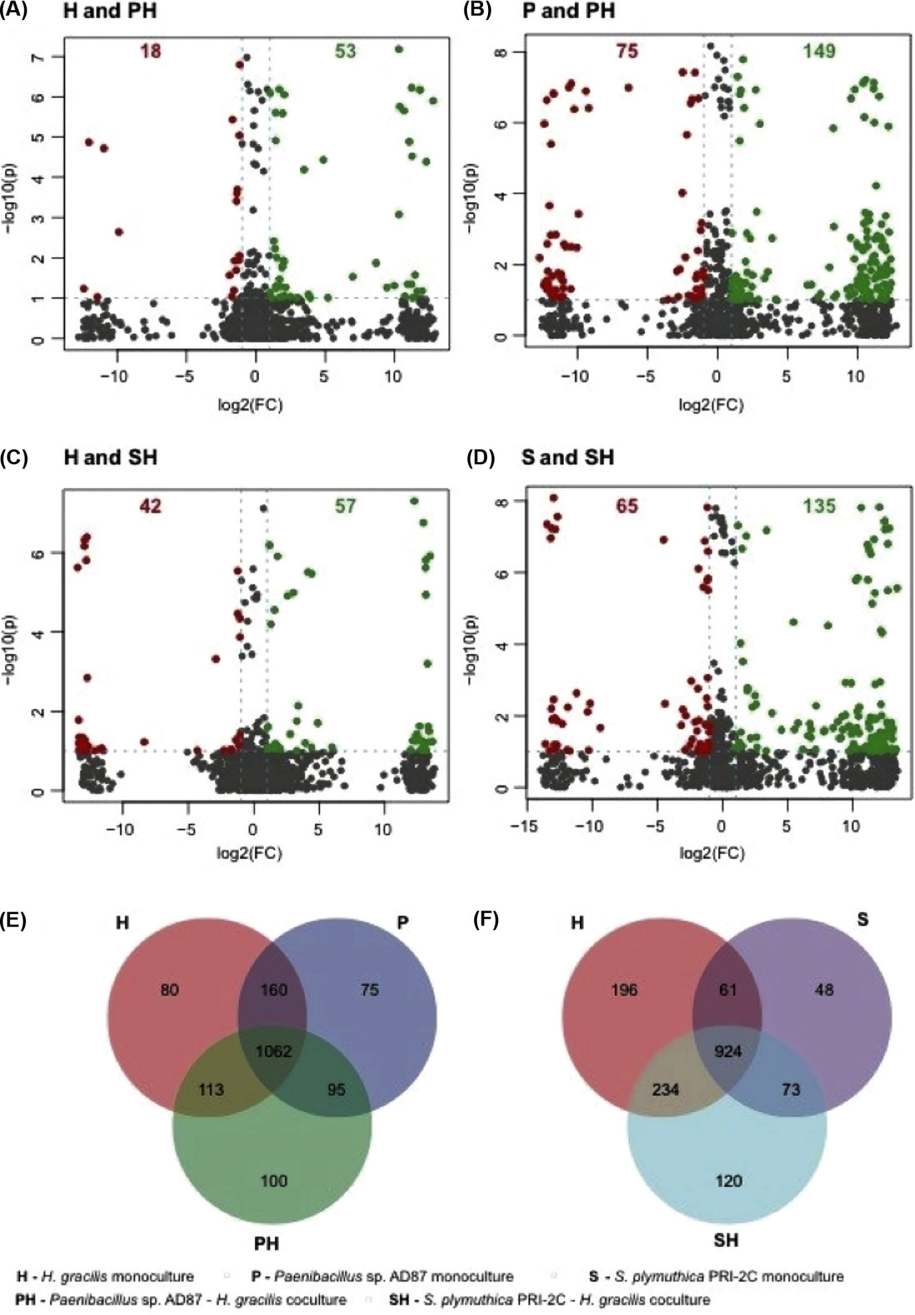
Volcano plots and Venn diagram to demonstrate metabolite concentration differences and unique/shared metabolites of the LAESI-MSI data. (A) Volcano plot for *H. gracilis* monoculture (H) versus *Paenibacillus* sp. AD87 with *H. gracilis* coculture (PH). (B) Volcano plot for *Paenibacillus* sp. AD87 monoculture (P) versus *Paenibacillus* sp. AD87 coculture with *H. gracilis* (PH). (C) Volcano plot for *H. gracilis* monoculture (H) versus *S. plymuthica* PRI-2C coculture with *H. gracilis* (SH). (D) Volcano plot for *S. plymuthica* PRI-2C monoculture (S) versus *S. plymuthica* PRI-2C coculture with *H. gracilis* (SH). Each point in the volcano plot represents one metabolite. Significantly differentially abundant metabolites were calculated with a fold change (FC) threshold of 2 on the *x* axis and a *t*-tests threshold of 0.1 on the *y* axis. The red and the green dots indicate statistically significant metabolites. The vertical FC threshold lines indicate an increase or decrease in concentration of metabolites. Negative log_2_ FC values indicated in red represent lower concentrations in native than in range-expanding species; positive values indicated in green represent higher concentrations of metabolites in native than in range-expanding species. (E) Venn diagram for H-P-PH. (F) Venn diagram for H-S-SH. To construct the Venn diagram, a single mass feature was considered even if it was present in only one replicate for a specific sample species.

Laser ablation electrospray ionization-mass spectrometry imaging (LAESI-MSI) was performed to visualize the localization of metabolites in their native environments in monoculture as well as during interaction without performing any extraction. Across all treatments, clear separation was observed among the samples for controls, monocultures, and interactions (Fig. 8A). An average of 1,050 mass features was detected per treatment. To list mass features that could explain separation among the controls, monocultures, and interactions, values of variable importance in projection (VIP) were calculated. The top 40 statistically significant mass features with VIP scores >2.0 are shown in Fig. 8B. The box-and-whisker plots for the four statistically significant differentially abundant metabolites selected from the volcano plot for the pair H and PH are shown in Fig. S2A. The volcano plot shown in Fig. 9A for *H. gracilis* monoculture (H) and the interaction of *H. gracilis* with *Paenibacillus* sp. AD87 (PH) shows 53 mass features (in green) located in the upper right quadrant, indicating that their concentrations are significantly higher in H compared to PH. Eighteen mass features (in red) in the upper left quadrant of the plot have a significantly lower concentration in H compared to PH. The ion intensity maps for these statistically significant metabolites are shown alongside box-and-whisker plots. The ion intensity maps are color coded based on the standard rainbow color scale where a pixel in red represented a high concentration and the pixel in black represents no concentration of the selected metabolite. As indicated, *m/z* 425.2886 and *m/z* 558.2832 show higher abundance in interaction sample PH, whereas *m/z* 410.8587 and *m/z* 716.7610 display high abundance in H compared to PH. For the pairwise analysis performed for *Paenibacillus* sp. AD87 monoculture (P) and the coculture of *H. gracilis* with *Paenibacillus* sp. AD87 (PH), 149 mass features (in green) displayed significantly high concentration in P and 75 mass features (in red) had significantly low concentration in P compared to PH (Fig. 9B). This is also evident in the box-and-whisker plots and the ion intensity maps that are presented for four statistically significant metabolites belonging to this set (Fig. S2B).

10.1128/msystems.00574-22.2 FIG S2 Box plots for the significantly differentially abundant metabolites and their corresponding ion intensity maps found using mass spectrometry imaging (MSI) during the interaction of *H. gracilis* with *Paenibacillus* sp. AD87 and *S. plymuthica* PRI-2C. Download FIG S2, PDF file, 0.6 MB. Copyright © 2022 Tyc et al. 2022 Tyc et al. https://creativecommons.org/licenses/by/4.0/ This content is distributed under the terms of the Creative Commons Attribution 4.0 International license.

For the pairwise analysis for the *H. gracilis* monoculture (H) and the coculture of *S. plymuthica* PRI-2C and *H. gracilis* (SH), 57 mass features (in green) displayed significantly high concentration in H and 42 mass features had significantly low concentration in HM compared to SH (Fig. 9C). The box-and-whisker plots along with the ion intensity maps for four statistically significant metabolites belonging to this set are shown in Fig. S2C. For the pairwise analysis for *S. plymuthica* PRI-2C monoculture (S) and the interaction of *S. plymuthica* PRI-2C and *H. gracilis* (SH), 135 mass features (in green) displayed significantly high concentration in S and 65 mass features had significantly low concentration in S compared to SH (Fig. 9D). The box-and-whisker plots along with the ion intensity maps for four statistically significant metabolites belonging to this set are shown in Fig. S2D.

To visualize the number of shared and unique metabolites among the monoculture and interaction samples, Venn diagrams were plotted. The Venn diagram (Fig. 9E) for monocultures *H. gracilis* and *Paenibacillus* sp. AD87 and their interaction shows 80 metabolites unique to *H. gracilis* monoculture, 75 metabolites unique to *Paenibacillus* sp. AD87 monoculture, and 100 metabolites that are unique during their interaction. A total of 1,062 metabolites were shared within these three treatments. Similarly, the Venn diagram (Fig. 9F) for monocultures *H. gracilis* and *S. plymuthica* PRI-2C and their interaction shows 196 metabolites unique to *H. gracilis* monoculture, 48 metabolites unique to *S. plymuthica* PRI-2C monoculture, and 120 metabolites that are unique during their interaction.

## DISCUSSION

Here, we report the first-time isolation of *H. gracilis* from a terrestrial soil sample. This bacterium passed a 0.1-μm filter, which suggests a very small cell size, theoretically justifying referring to these bacteria as ultrasmall bacteria (26). However, against our expectation, the microscopic analysis revealed that this bacterium is not ultrasmall in cell size but possesses a very thin diameter and showed the typical spiraled morphology known for these species (38–41). These observations are in line with previous research by Wang et al. (42) showing that *H. gracilis* can pass through filters of various pore sizes ranging from 0.45 μm to 0.1 μm, most probably thanks to their cell shape and cell morphology. *In silico* analysis of 16 terrestrial metagenome data available on MG-RAST (https://www.mg-rast.org/) showed that *H. gracilis* was not present in terrestrial metagenome data (not shown), suggesting that *H. gracilis* is not commonly present in terrestrial soils. The bacterial interaction assays revealed that *H. gracilis* grows better when interacting with *Paenibacillus* sp. AD87 or *S. plymuthica* PRI-2C. The cell numbers of *H. gracilis* were higher when exposed to cell-free supernatants of *Paenibacillus* sp. AD87 and *S. plymuthica* PRI-2C, suggesting that the metabolites released by the latter bacteria in cocultures with *H. gracilis* are associated with improved growth of *H. gracilis*. We hypothesized that *H. gracilis* grows better in coculture, either because growth is stimulated by signals produced by the other organism, or because the environment that is created by the other organism allows *H. gracilis* to make more efficient use of certain metabolic pathways. Indeed, the metabolic experiments with Biolog plates showed that during interspecific interactions of *H. gracilis* with *Paenibacillus* sp. AD87 or with *S. plymuthica* PRI-2C, more carbohydrates could be utilized compared to the monocultures. This is an interesting observation, and it may indicate that interaction of bacteria can trigger the production of exoenzymes, enabling the degradation of carbohydrates, which the bacteria were not able to degrade in monoculture.

We speculated that since *H. gracilis* grows better in interaction with other bacteria and is of relatively small cell size, *H. gracilis* might have evolved according to a genome streamlining strategy, i.e., the adaptive loss of genes for which functions it relies on interaction with other bacteria in the immediate environment. The whole-genome sequencing of *H. gracilis* revealed a genome size of 3.82 Mbp. This is a relatively small genome size for free-living soil bacteria that typically have estimated average genome sizes of ~4.7 Mbp (36, 43–46). The *in silico* antiSMASH (37) comparison of genes that are part of secondary metabolite gene clusters showed that the *H. gracilis* genome contained only three gene clusters encoding the production of secondary metabolites (bacteriocins, terpenes, and aryl polyenes). Terpenes and aryl polyenes are known as protective compounds against abiotic stressors, while bacteriocins have antimicrobial activities against closely related bacteria (17, 47–51). We hypothesize that *H. gracilis* genome streamlining has allowed it to be more competitive, by retaining only the most essential metabolic functions while having roughly about one quarter less DNA to replicate during each cell division. Gene loss and reduced genome size may cause dependency on other microbes in their surroundings, and this may explain a considerable part of the phenomenon that most of the detectable bacteria in the environment are not cultivable under laboratory conditions.

To investigate the mechanisms of interaction, we performed transcriptome analysis on the interaction pairs of *H. gracilis* with *S. plymuthica* PRI-2C and *Paenibacillus* sp. AD87. Interestingly, a larger amount of significantly differentially expressed genes was induced by *H. gracilis* in the other two competing bacteria compared to the transcriptomic changes in *H. gracilis.* Several processes, enriched according to GO term enrichment analysis, could be part of a mechanism(s) mediating interactions between *H. gracilis* and *S. plymuthica* PRI-2C and *Paenibacillus* sp. AD87, for example genes related to chemotaxis. Moreover, the GO terms for signal transduction, secondary metabolite production, and cell motility were enriched in the transcriptome of *H. gracilis* during the cocultivation with *Paenibacillus* sp. AD87, suggesting that chemotaxis and cell movement is an important feature during interspecific interactions between these two bacterial taxa (52, 53). In addition, GO terms referring to iron-sulfur (Fe-S) complex assembly were enriched in the transcriptomes of *H. gracilis* during the cocultivation with *S. plymuthica* PRI-2C and *Paenibacillus* sp. AD87. Fe-S clusters are important for sustaining fundamental life processes: they participate in electron transfer, substrate binding/activation, iron or sulfur storage, regulation of gene expression, and enzyme activity (54, 55). This upregulation could indicate that, potentially, in coculture, normal-sized bacteria released metabolites that *H. gracilis* used for synthesizing Fe-S complexes. It is also possible that iron-sulfur complex assembly is activated during competition with the interacting bacteria for sulfur or iron collection (scavenging) (56–59).

The metabolic pathway analysis showed that the loss of genes in *H. gracilis* does not appear to have resulted in functional loss of metabolic pathways. Loss of nonessential and possibly redundant genes in several metabolic pathways could explain why and how the genome of *H. gracilis* has become so small. The missing genes are not essential to complete metabolic pathways and only appear to result in limited options in certain metabolic pathways. RAST analysis showed that all basal metabolic pathways remain feasible with the annotated enzymes and pathways of *H. gracilis*. The only exception is EC term 5.2.1.1 (maleate isomerase). There are several ways to synthesize fumarate, e.g., in the glycolysis pathway (41, 60, 61) and in the citric acid cycle (41, 62). Based on the available data, it cannot be unambiguously determined which alternative pathway may preferably be used by *H. gracilis* to synthesize fumarate.

The metabolomics analysis revealed the production of specific antimicrobial compounds such as pyrollnitrin (*S. plymuthica* PRI-2C) and 2,5-bis(1-methylethyl)-pyrazine (*Paenibacillus* sp. AD87) which are well known for their broad-spectrum antimicrobial activity (63–67). However, the produced antimicrobial compounds didn’t show activity against *H. gracilis*: in both interactions, *H. gracilis* showed increased growth when growing in coculture with either *Paenibacillus* sp. AD87 or *S. plymuthica* PRI-2C.

The understanding of natural metabolites that mediate interactions between organisms in natural environments is the key to elucidate ecosystem functioning. The detection and identification of the compounds that mediate such interactions is still challenging. Techniques such as mass spectrometry imaging (MSI) provide new opportunities to study environmentally relevant metabolites in their spatial context (68–70). In this study, the metabolomics was performed using three independent approaches namely, DART-MS analysis, GC/MS-Q-TOF analysis and LAESI-MSI from living bacterial colonies. LAESI-MSI analysis revealed that several mass features were detected in higher abundance during the cocultivation of *H. gracilis* with *Paenibacillus* sp. AD87, these mass features were *m/z* 425.2886 and *m/z* 558.2832. LAESI-MSI is not suitable for unambiguous compound annotation, but can still be used for putative compound annotation. To annotate the detected mass features to compounds with high certainty, LAESI-MSI should be coupled with ion mobility separation as previously suggested (71–73). Yet, LAESI-MSI can help to spatially distinguish the produced secondary metabolites of living bacterial colonies with limited sample preparation and can give insight into the spatial distribution of metabolites.

Several studies indicate that the volatile blend composition of the volatiles greatly depends on biotic interactions and on growth conditions (15, 19, 74–76). Here, a higher number of volatile compounds were detected in the bacterial cocultures, most likely due to the combination of emitted volatiles of the interacting bacteria. The high number of sulfur-containing compounds indicates that these compounds are commonly produced by bacteria and might play an important role in signaling during interspecific interactions (77, 78). No novel volatile compounds were detected during the coculture of the three bacteria.

Overall, our study showed that *H. gracilis* can pass through a 0.1-μm filter and is present in terrestrial environments. The growth performance and physiological behavior of *H. gracilis* were dependent on the cocultivated bacterial partner and they might be metabolically depending on the cocultivated bacteria. At the same time, *H. gracilis* was able to change the physiology, release of volatile organic compounds, and secreted enzymes of the cocultivated bacteria without direct cell-cell contact.

Microbial interspecific interactions play an important role in the functioning of the terrestrial ecosystem. Soil microbial communities are very diverse and dynamic and involve frequent and sporadic interspecific interactions. Our study indicates that sulfur and Fe-S clusters could play an important role in microbial interspecific interactions in terrestrial environments and more studies are required to understand their role. The study of sporadic interspecific interactions and the inclusion of rare taxa in future analysis could help to better understand microbial communities and functions of those.

## MATERIALS AND METHODS

### Isolation and identification of bacteria that pass through 0.1-μm filters.

**(i) Isolation of *H. gracilis* from soil.** After removing the grassland vegetation, a topsoil core was collected and mixed; a sample of 10 g was suspended in 90 mL of 10 mM phosphate-buffer (pH 6.5) and shaken with gravel (2 to 4 mm) at 250 rpm for 45 min. The extract was filtered through sterile gauze pads and subsequently through sterile Whatmann 1-mm paper filter using Buchner funnel. Purified extract was filtered again through a syringe filter (0.2 μm) and afterwards through a 0.1-μm filter (GE-Healthcare). The filtered extract was plated on 1/10th tryptic soy broth agar (TSBA) plates immediately after isolation and incubated at 25°C (Fig. S1). The plates were inspected daily using a stereomicroscope (Leica M205C) screening for bacteria colonies.

10.1128/msystems.00574-22.1 FIG S1 Schematic overview of the applied isolation method used to isolate *H. gracilis* from soil. Download FIG S1, PDF file, 0.1 MB. Copyright © 2022 Tyc et al. 2022 Tyc et al. https://creativecommons.org/licenses/by/4.0/ This content is distributed under the terms of the Creative Commons Attribution 4.0 International license.

**(ii) Bacterial and culture conditions.** The bacterial strains used in this study are the Gram-negative strain *S. plymuthica* PRI-2C (*Gammaproteobacteria*) (79), the Gram-positive strain *Paenibacillus* sp. AD87 (*Firmicutes*) (10, 80, 81), and the Gram-negative *H. gracilis* isolate NS1 (*Betaproteobacteria*). The bacterial isolates were precultured from −80°C glycerol stocks on 1/10th TSBA (82) or on Luria-Bertani agar (LB-A) (*H. gracilis*) (LB-Medium Lennox, Carl Roth GmbH + Co. KG, 20 gL^−1^ Bacto Agar) and incubated at 24°C prior application.

**(iii) Identification of *H. gracilis.*** For the identification of *H. gracilis*, 16S rRNA PCR was performed from grown colonies in a 50-μL PCR-GoTaq green master mix (Promega Corp. Madison, USA; cat. no. M712). For 16S rRNA gene amplification, the following primers were used: forward primer 27f (5′-AGA GTTT GAT CMT GGC TCAG-3′), reverse primer 1492r (5′-GRT ACC TTG TTA CGA CTT-3′), amplifying ~1,465 bp from the 16S rRNA gene (83, 84) (modified). All PCRs were performed on a Bio-Rad C1000 Touch PCR machine (BIO-RAD, Veenendaal, the Netherlands) with these settings: initial cycle of 95°C for 3 min; 30 cycles of 94°C for 30 sec, 55°C for 45 sec, and 72°C for 1 min; and final extension at 72°C for 5 min. The PCR products were purified using the Qiagen PCR purification kit and sent to MACROGEN (MACROGEN Europe, Amsterdam, the Netherlands) for 16S rRNA sequencing.

**(iv) Microscopy.** Microscopy pictures of *H. gracilis* cells were taken at 400-fold magnification with an Axio Imager M1 microscope (Carl Zeiss, Germany) under phase-contrast illumination with an AxioCam MRm camera. Macroscopic colony pictures of *H. gracilis* were taken with an Olympus Binocular at ×20 magnification. Images were analyzed with AxioVision v4.7 (Carl Zeiss Imaging Solutions GmbH, Germany).

**(v) Bacterial interactions assays.** After four days of preculture, a single colony of *Paenibacillus* sp. AD87 and *S. plymuthica* PRI-2C and *H. gracilis* was picked and inoculated in 20 mL 1/10th TSB (*Paenibacillus* sp. AD87 and *S. plymuthica* PRI-2C) and grown overnight at 24°C, 220 rpm. For the inoculation of *H. gracilis*, a single colony was picked from a TSBA plate and inoculated in 20 mL LB-medium and grown for 3 days at 24°C, 200 rpm. For the interaction assay, an inoculation mix of each bacterial strain (*Paenibacillus* sp. AD87, *S. plymuthica* PRI-2C, and *H. gracilis*) was prepared by diluting the bacterial isolates in 20 mL of 10 mM phosphate-buffer (pH 6.5) to an optical density at 600 nm (OD_600_) of 0.005 (*Paenibacillus* sp. AD87 and *S. plymuthica* PRI-2C) or to an OD_600_ of 0.05 (*H. gracilis*), which corresponds to 10^5^ CFU/mL. A droplet of 10 μL inoculation mixture was added in the middle of a 6-cm diameter petri dish (monocultures) or next to each other in a distance of ~0.5 cm (pairwise interactions). All treatments were performed in triplicates on 1/10th TSBA plates incubated at 24°C. After the growth time of 3 days, the bacteria were scratched and washed from the plates by using sterile cell scratchers and Phosphate buffer. For the enumeration of the cell counts (CFU/mL), dilution series of the scratched bacteria were prepared and plated in triplicates on 1/10th TSBA plates and grown for 48 h. Enumeration was carried out on an aCOlyte colony counter (Don Whitley Scientific, Meintrup DWS Laborgeräte GmbH, Germany) based on their different morphology.

### Enumeration of growth-inhibitory or growth-promoting effects of cell-free supernatants of *Paenibacillus* sp. AD87and *S. plymuthica* PRI-2C on the growth of *H. gracilis*.

A bacterial growth assay in liquid media supplemented with cell-free supernatants (CFS) of *Paenibacillus* sp. AD87 and *S. plymuthica* PRI-2C was conducted. For the assay, single colonies of *Paenibacillus* sp. AD87, *S. plymuthica* PRI-2C, and *H. gracilis* were inoculated in 20 mL 1/10th TSB (*Paenibacillus* sp. AD87 and *S. plymuthica* PRI-2C) or in 20 mL LB medium (*H. gracilis*) and grown overnight (*Paenibacillus* sp. AD87 and *S. plymuthica* PRI-2C) or for 3 days (*H. gracilis*) at 24°C, 220 rpm. For the preparation of *Paenibacillus* sp. AD87 and *S. plymuthica* PRI-2C CFS, the grown cultures were centrifuged at 5,000 rpm for 20 min (at room temperature) and filtered through 0.2-μm filters (GE Healthcare). For the assay, *H. gracilis* was inoculated into 20 mL liquid LB media at an OD_600_ of 0.05. The growth medium was then supplemented either with 20% (vol/vol) CFS of *Paenibacillus* sp. AD87 or *S. plymuthica* PRI-2C or with 20% (vol/vol) of filter-sterilized liquid 1/10th TSB media (control). The cultures were incubated at 24°C at 220 rpm for 7 days and the bacterial growth was monitored by optical density (absorbance at 600 nm) measurements and by plate counting. After 5 days of growth, the CFU/mL of *H. gracilis* grown in the presence of CFS of *Paenibacillus* sp. AD87 or *S. plymuthica* PRI-2C were enumerated by plate counting. For this, the cultures were sampled, and dilution series were prepared in triplicates and a volume of 100 μL of each serial dilution was plated in three replicates with a disposable Drigalski spatula on 1/10th TSBA plates. CFU Enumeration was carried out on an aCOlyte colony counter.

### DNA isolation and genome sequencing of *H. gracilis*.

Genomic DNA of *H. gracilis* was extracted using a Qiagen genomic-tip 500/G DNA kit (Qiagen, cat. no. 10262). Genome sequencing was performed on the PacBio RS II platform (Pacific Biosciences, Menlo Park, CA, USA) using P6-C4 chemistry at the Institute for Genome Sciences (IGS), Baltimore, MD, USA. The sequencing resulted in a total of 70,101 reads with *N*_50_ of 17,309 nucleotides. The PacBio raw sequences were analyzed using SMRT portal v2.3.0.140936 p.4150482. Sequences were assembled *de novo* with the RS_HGAP_assembly 3 software (Pacific Biosciences) with default settings on an estimated genome size of 3.8 Mbp. The resulting assemblies were subjected to scaffolding using the RS_AHA_scaffolding 1 software. The genome assembly properties are shown in Table 1. Final contigs were annotated using PROKKA v1.11 (85) and InterproScan v5.16 55.0 (86). The whole-genome sequence was submitted as Hylemonella gracilis strain NS1 to NCBI GenBank (https://www.ncbi.nlm.nih.gov/genbank/) under accession no. CP031395.

### *In silico* analysis of secondary metabolite gene clusters.

For *in silico* analysis of secondary metabolite gene clusters, the genome sequences of *H. gracilis*, *Paenibacillus* sp. AD87, and *S. plymuthica* PRI-2C were submitted to the antiSMASH web server (http://antismash.secondarymetabolites.org/) v4.0 (37).

### RNA isolation and sequencing.

Sampling for total RNA extractions was performed in triplicates after five and 10 days of incubation on bacteria grown on 1/10th TSBA plates either in coculture or monoculture as described previously (bacterial interactions assays on 1/10th TSBA plates). For the isolation of bacterial cell material, a volume of 1 mL of 10-mM phosphate buffer (pH 6.5) was added to the surface of the 1/10th TSBA plates and grown bacterial cells were suspended from the plate surface with a disposable cell scratcher (VWR International B.V., the Netherlands). For total RNA extraction the obtained cell suspension was transferred to a tube containing RNAprotect bacteria reagent (Qiagen, cat. no. 76506) and centrifuged for 20 min at 20,000 *g*, 4°C. The supernatant was discarded, and the resulting cell pellets were stored at −80°C. Total RNA was extracted using the Aurum Total RNA minikit (Bio-Rad) according to the manufacturer’s protocol. Samples were treated with the Ambion TURBO DNA free kit according to the manufacturer’s protocol. The RNA concentration and quality was checked on a NanoDrop Spectrophotometer (ND 2000, Thermo Fisher Scientific, the Netherlands) and on a 1.0% Tris-borate-EDTA (TBE) agarose gel. Samples were subjected to RNA sequencing at the Erasmus Center for Biomics (www.biomics.nl), Erasmus MC, Rotterdam, the Netherlands using the Illumina HiSeq 2500 sequencing platform. The obtained reads were checked for quality using Fastq. For the estimation of the transcripts, the filtered sequences were aligned against the cDNA sequences of *H. gracilis*, *Paenibacillus* sp. AD87 and *S. plymuthica* PRI-2C using Bowtie 2 (v2.2.5) (87) with the following settings: – no-mixed – no-discordant – gbar 1000 – end-to-end. Transcript abundance was calculated using RSEM v1.1.26 (88) and differential expression between the treatments was calculated using the edgeR v3.2 package in the R environment (89–91).

### Pathway annotations.

Please see Supplemental Methods (Text S1).

### Exploration of missing genes and genome streamlining in *Hylemonella*.

RAST annotations of *S. plymuthica* PRI-2C, *Paenibacillus* sp. AD87, and *H. gracilis* were used to compare their genomes and to explore the genomes for missing genes in metabolic pathways (http://rast.nmpdr.org) (92–94). The missing gene sequences were extracted and assigned with KEGG orthology (95, 96). Presence/absence of genes belonging to metabolic pathways was compared across the three genomes to identify shared genes and pathways and to determine incomplete metabolic pathways in *H. gracilis*.

### Catabolic profiling.

To determine the carbon source usage abilities of *H. gracilis* and *Paenibacillus* sp. AD87 and *S. plymuthica* PRI-2C strains, Biolog EcoPlate (Labconsult S.A.-N.V., Brussels, Belgium) assays were performed (97, 98). Bacteria were cultured in monoculture or in coculture in single wells of the Biolog EcoPlate. One single colony of each bacterial strain was picked and inoculated in 15 mL 1/10th TSB or 15 mL LB liquid media. Bacteria were grown overnight (*Paenibacillus* sp. AD87 and *S. plymuthica* PRI-2C) or for 2 days (*H. gracilis*) at 24°C at 250 rpm. Grown bacterial cultures were washed twice by centrifugation at 4.500 rpm for 15 min at room temperature, the supernatant was discarded, and the pellet was washed and resuspended in 5 mL of 10 mM phosphate buffer. The reuspended cultures were diluted to an OD_600_ of 0.005 in 20 mL of 10-mM phosphate buffer either in monoculture or in coculture. For the experiment, Biolog EcoPlates were inoculated with 100 μL of each bacterial inoculation suspension (monocultures or cocultures) in each well. For each bacterial monoculture one Biolog EcoPlate was inoculated, as well for each cocultivation pair (*S. plymuthica* PRI-2C with *H. gracilis* and *Paenibacillus* sp. AD87 with *H. gracilis*). Plates were incubated for 1 week and absorbance was measured at 590 nm every 24 h on a BIOTEK plate reader to determine the ability of the bacterial cultures to use the carbon sources present in the wells.

### Trapping of volatile organic compounds and GC-Q-TOF analysis.

Please see Supplemental Methods (Text S1).

### DART-MS data analysis.

Please see Supplemental Methods (Text S1).

### Ambient mass-spectrometry imaging LAESI-MS data analysis.

For LAESI-MS analysis, a single colony of each bacterial isolate was picked and inoculated in 20 mL 1/10th TSB (*Paenibacillus* sp. AD87 and *S. plymuthica* PRI-2C) and grown overnight at 24°C at 220 rpm. For the inoculation of *H. gracilis*, a single colony was picked from plate and inoculated in 20 mL LB medium and grown for 3 days at 24°C at 200 rpm. The inoculation mixture was prepared by diluting the bacterial isolates in 20 mL of 10 mM phosphate buffer (pH 6.5) to an OD_600_ of 0.005. The inoculation mixture was pulse-vortexed for 30 sec and a droplet of 10 μL was added in the middle of a 6-cm diameter petri dish (monocultures) or next to each other in a distance of approximately 0.5 cm (pairwise interactions). All treatments were inoculated in triplicates on 1/10th TSBA and incubated at 24°C for five and 10 days. After five and 10 days of incubation, bacterial colonies were cut out of the agar (size approximately 1 to 3 cm^2^) and subjected to LAESI-MS measurement. The LAESI-MS analysis was carried out on a Protea Biosciences DP-1000 LAESI system (Protea Bioscience Inc., Morgantown, WV, USA) coupled to a Waters model Synapt G2S (Waters Corporation, Milford, MA, USA) mass spectrometer. The LAESI system was equipped with a 2940-nm midinfrared laser yielding a spot size of 100 μm. The laser was set to fire 10 times per x-y location (spot) at a frequency of 10 Hz and 100% output energy. A syringe pump was delivering the solvent mixture of methanol/water/formic-acid (50:50:0.1% vol/vol) at 2 μL/min to a PicoTip (5 cm × 100 μm diameter) stainless steel nanospray emitter operating in positive ion mode at 4,000 V. The LAESI was operated using LAESI desktop software v2.0.1.3 (Protea Biosciences Inc., Morgantown, WV, USA). The time of flight (TOF) mass analyzer of the Synapt G2S was operated in the V-reflectron mode at a mass resolution of 18.000 to 20.000. The source temperature was 150°C, and the sampling cone voltage was 30 V. The positive ions were acquired in a mass range of 50 to 1,200 *m/z*. The MS data were lock mass corrected post data acquisition using leucine encephalin (C_25_H_37_N_5_O_7_ *m/z* = 556.2771), which was used as an internal standard. All the acquired Waters *.RAW data files were converted to open file format *.imzML using an in-house script written in R. Later, these data were preprocessed in multiple steps to remove noise and to make the data comparable. First, square root transformation was applied to the data to stabilize the variance. Then, baseline correction was performed to enhance the contrast of peaks to the baseline. For better comparison of intensity values and to remove small batch effects, Total Ion Current (TIC)-based normalization was applied. This was followed by spectral alignment and peak detection to extract a list of significant mass features for each sample replicate per treatment. In the end, a mass feature matrix was generated with sample replicates for each treatment in columns and mass features in rows. This feature matrix was used to perform further statistical analysis. The preprocessing and peak-detection steps were applied using R scripts developed in-house and using functions available within the MALDIquant R package (99). To perform multivariate analysis, the feature matrix was imported into the online version of Metaboanalyst 4.0 (100). Ion intensity maps displaying the spatial distribution for statistically significant mass features were created using R. Before generating the ion maps, the intensity values for the selected mass features were normalized to the maximum intensity within the image, measured for each mass value individually. Venn diagrams displaying unique and common masses among different treatments were drawn using the jvenn tool (101).

### Data availability.

The raw data of this article will be made available by the authors to any qualified researcher upon request. The whole-genome sequence of Hylemonella gracilis strain NS1 is available at the NCBI GenBank (https://www.ncbi.nlm.nih.gov/genbank/) under accession no. CP031395, and the raw reads of the transcriptomics data are available at the Sequence Read Archive (SRA) (https://www.ncbi.nlm.nih.gov/sra) under accession no. PRJNA483535. The metabolomics raw data (DART-MS and LAESI-MSI) are available at metabolights (www.ebi.ac.uk/metabolights/MTBLS5841).

10.1128/msystems.00574-22.8 TABLE S6 Significantly up- or downregulated genes of *H. gracilis* responding to *Paenibacillus* sp. AD87 at day 5. Download Table S6, PDF file, 0.08 MB. Copyright © 2022 Tyc et al. 2022 Tyc et al. https://creativecommons.org/licenses/by/4.0/ This content is distributed under the terms of the Creative Commons Attribution 4.0 International license.

10.1128/msystems.00574-22.9 TABLE S7 Significantly up- or downregulated genes of *Paenibacillus* responding to *H. gracilis* at day 10. Download Table S7, PDF file, 0.08 MB. Copyright © 2022 Tyc et al. 2022 Tyc et al. https://creativecommons.org/licenses/by/4.0/ This content is distributed under the terms of the Creative Commons Attribution 4.0 International license.

10.1128/msystems.00574-22.10 TEXT S1 Supplemental methods. Pathway annotations. GC-Q-TOF data analysis. Direct Analysis in Real Time Mass Spectrometry (DART-MS) data analysis. Download Text S1, DOCX file, 0.02 MB. Copyright © 2022 Tyc et al. 2022 Tyc et al. https://creativecommons.org/licenses/by/4.0/ This content is distributed under the terms of the Creative Commons Attribution 4.0 International license.
