# Supplementary material for: Exploring the Interspecific Interactions and the Metabolome of the Soil Isolate Hylemonella gracilis
Source: mSystems. 2022 Dec 20;8(1):e00574-22. doi: 10.1128/msystems.00574-22 (PMC9948732; doi:10.1128/msystems.00574-22)
Supplement: TABLE S1 [file msystems.00574-22-s0004.pdf]

Supplementary Table 1: Significantly differentially expressed genes of *Paenibacillus* sp. AD87, responding to *H. gracilis* at day 10.

Gene	logFC	PValue	FDR	Function
gpAD87_RS06700	-2.9328905	8.66E-15	6.48E-11	rpoE; RNA polymerase sigma-70 factor, ECF subfamily
gpAD87_RS13150	-1.8451399	7.78E-05	0.04477567	opuBD; osmoprotectant transport system permease protein
gpAD87_RS13150	-1.8451399	7.78E-05	0.04477567	opuC; osmoprotectant transport system substrate-binding protein
gpAD87_RS30390	-1.6913798	2.06E-06	0.0030772	tatD; TatD DNase family protein [EC:3.1.21.-]
gpAD87_RS19945	-1.2794042	1.17E-05	0.01253836	UXS1; UDP-glucuronate decarboxylase [EC:4.1.1.35]
gpAD87_RS00275	-1.2612217	1.63E-05	0.01628367	E2.2.1.2; transaldolase [EC:2.2.1.2]
gpAD87_RS19920	-1.2608789	1.78E-05	0.0166361	pimB; phosphatidyl-myo-inositol dimannoside synthase [EC:2.4.1.346]
gpAD87_RS00270	-1.2512648	5.28E-05	0.03593459	PGD; 6-phosphogluconate dehydrogenase [EC:1.1.1.44 1.1.1.343]
gpAD87_RS19895	-1.1972691	4.98E-06	0.00620873	UGDH; UDPglucose 6-dehydrogenase [EC:1.1.1.22]
gpAD87_RS21205	-1.1167672	2.65E-06	0.00360466	N/A
gpAD87_RS26695	-0.9979737	3.18E-05	0.02642266	gerKA; spore germination protein KA
gpAD87_RS00725	1.0213585	9.92E-06	0.01142133	E3.1.3.15B; histidinol-phosphatase (PHP family) [EC:3.1.3.15]
gpAD87_RS17335	1.43673471	9.05E-05	0.0483872	abrB; transcriptional pleiotropic regulator of transition state genes
gpAD87_RS10500	1.48682555	6.63E-05	0.04132522	deoC; deoxyribose-phosphate aldolase [EC:4.1.2.4]
gpAD87_RS11290	1.69953743	1.47E-06	0.00243757	folD; methylenetetrahydrofolate dehydrogenase (NADP+)
