# Supplementary material for: Exploring the Interspecific Interactions and the Metabolome of the Soil Isolate Hylemonella gracilis
Source: mSystems. 2022 Dec 20;8(1):e00574-22. doi: 10.1128/msystems.00574-22 (PMC9948732; doi:10.1128/msystems.00574-22)
Supplement: TABLE S2 [file msystems.00574-22-s0005.pdf]

Supplementary Table 2: Significantly differentially expressed genes of *Serratia plymuthica* PRI-2C responding to *H. gracilis* at day 5.

Gene	logFC	PValue	FDR	Function
Q5A_025180	1.484670421	4.37298312	1.16E-05	rph; ribonuclease PH [EC:2.7.7.56]
