# Supplementary material for: Exploring the Interspecific Interactions and the Metabolome of the Soil Isolate Hylemonella gracilis
Source: mSystems. 2022 Dec 20;8(1):e00574-22. doi: 10.1128/msystems.00574-22 (PMC9948732; doi:10.1128/msystems.00574-22)
Supplement: TABLE S3 [file msystems.00574-22-s0006.pdf]

Supplementary Table 3: Significantly differentially expressed genes of *Serratia plymuthica* PRI-2C responding to *H. gracilis* at day 10.

Gene	logFC	PValue	FDR	Function
Q5A_020525	-3.539216745	3.79E-13	2.98E-10	hcp; type VI secretion system secreted protein Hcp
Q5A_020555	-2.840557251	6.03E-06	0.0011281	fimA; major type 1 subunit fimbrin (pilin)
Q5A_023300	-2.169738036	6.89E-08	2.10E-05	malK; multiple sugar transport system ATP-binding protein [EC:3.6.3.-]
Q5A_011700	-1.997102593	1.42E-05	0.0021876	ynfM; MFS transporter, YNFM family, putative membrane transport protein
Q5A_003445	-1.972950006	1.13E-06	0.00024804	garD; galactarate dehydratase [EC:4.2.1.42]
Q5A_018430	-1.744985992	9.02E-05	0.00993022	N/A
Q5A_009750	-1.734981082	4.74E-05	0.00622171	ftnA; ferritin [EC:1.16.3.2]
Q5A_020535	-1.729517104	0.000274679	0.02461418	impB; type VI secretion system protein ImpB
Q5A_023295	-1.625377127	2.34E-10	1.17E-07	lamB; maltoporin
Q5A_003465	-1.528329707	2.21E-05	0.00327673	garL; 2-dehydro-3-deoxyglucarate aldolase [EC:4.1.2.20]
Q5A_003455	-1.527498481	8.21E-05	0.00952465	gudX; glucarate dehydratase-related protein
Q5A_003450	-1.467669658	3.67E-06	0.00074239	gudP; MFS transporter, ACS family, glucarate transporter
Q5A_010395	-1.445444456	9.84E-06	0.00165079	puuD; gamma-glutamyl-gamma-aminobutyrate hydrolase [EC:3.5.1.94]
Q5A_020530	-1.425017256	5.82E-05	0.00719325	impC; type VI secretion system protein ImpC
Q5A_002075	-1.344637355	0.000219471	0.02052737	ABC-2.P; ABC-2 type transport system permease protein
Q5A_015990	-1.328859825	1.07E-07	3.15E-05	paaC; ring-1,2-phenylacetyl-CoA epoxidase subunit PaaC [EC:1.14.13.149]
Q5A_010390	-1.305376856	1.67E-07	4.46E-05	puuR; HTH-type transcriptional regulator, repressor for puuD
Q5A_023310	-1.296519929	5.59E-09	1.99E-06	malE; maltose/maltodextrin transport system substrate-binding protein
Q5A_005185	-1.29424411	1.11E-07	3.19E-05	cybB; cytochrome b561
Q5A_003460	-1.287052853	0.000331618	0.02833289	gudD; glucarate dehydratase [EC:4.2.1.40]
Q5A_015985	-1.157880882	1.10E-05	0.00177766	paaB; ring-1,2-phenylacetyl-CoA epoxidase subunit PaaB
Q5A_009735	-1.129234286	2.54E-08	8.63E-06	dksA; DnaK suppressor protein
Q5A_016000	-1.118109907	1.34E-05	0.0021073	paaE; ring-1,2-phenylacetyl-CoA epoxidase subunit PaaE
Q5A_015980	-1.11728173	4.91E-06	0.00094221	paaA; ring-1,2-phenylacetyl-CoA epoxidase subunit PaaA [EC:1.14.13.149]
Q5A_017330	-1.103510732	4.63E-06	0.00091123	katE; catalase [EC:1.11.1.6]
Q5A_015995	-1.101590323	7.03E-06	0.00123738	paaD; ring-1,2-phenylacetyl-CoA epoxidase subunit PaaD
Q5A_015975	-1.093213554	3.94E-06	0.00078613	paaZ; oxepin-CoA hydrolase / 3-oxo-5,6-dehydrosuberyl-CoA semialdehyde dehydrogenase
Q5A_016010	-1.074845013	1.36E-05	0.00212107	paaG; 2-(1,2-epoxy-1,2-dihydrophenyl)acetyl-CoA isomerase [EC:5.3.3.18]
Q5A_013825	-1.003817338	0.000406824	0.03308758	osmB; osmotically inducible lipoprotein OsmB
Q5A_008760	-0.988582472	0.000254571	0.02322956	hspQ; heat shock protein HspQ
Q5A_009380	-0.977583963	0.000538345	0.04089512	bssS; biofilm regulator BssS
Q5A_006035	-0.974655768	1.64E-05	0.00249851	acpD; FMN-dependent NADH-azoreductase [EC:1.7.-.-]
Q5A_003705	-0.961603947	0.000667859	0.04823509	csrA; carbon storage regulator
Q5A_005195	-0.95850798	1.04E-05	0.00169051	tomB; hha toxicity modulator TomB
Q5A_004910	-0.924784476	1.74E-05	0.00262875	panE; 2-dehydropantoate 2-reductase [EC:1.1.1.169]
Q5A_011805	-0.908861788	0.000201997	0.01937745	xdhB; xanthine dehydrogenase large subunit [EC:1.17.1.4]
Q5A_005190	-0.887772818	0.00022171	0.02060805	hha; haemolysin expression modulating protein
Q5A_015190	-0.886911712	0.000277108	0.02468407	phoH; phosphate starvation-inducible protein PhoH and related proteins
Q5A_013870	-0.881053434	0.000295228	0.0259887	ribA; GTP cyclohydrolase II [EC:3.5.4.25]
Q5A_010385	-0.872345769	7.46E-05	0.00892893	puuC; 4-(gamma-glutamylamino)butanal dehydrogenase [EC:1.2.1.99]
Q5A_024620	-0.869220332	0.000360469	0.03030572	membrane protein
Q5A_001015	-0.867584758	0.000474421	0.03697766	livM; branched-chain amino acid transport system permease protein
Q5A_023890	-0.838466925	0.000467223	0.0367344	PPIA; peptidyl-prolyl cis-trans isomerase A (cyclophilin A) [EC:5.2.1.8]
Q5A_022860	-0.833565851	0.000526905	0.04023029	yhcO; ribonuclease inhibitor
Q5A_017850	-0.83112707	0.000165885	0.01633202	mlaA; phospholipid-binding lipoprotein MlaA
Q5A_022140	-0.824798608	0.000584632	0.04357256	dkgA; 2,5-diketo-D-gluconate reductase A [EC:1.1.1.346]
Q5A_014735	-0.785920924	0.000231103	0.02134478	PTS-Man-EIIB; PTS system, mannose-specific IIB component [EC:2.7.1.191]
Q5A_014735	-0.785920924	0.000231103	0.02134478	PTS-Man-EIIA; PTS system, mannose-specific IIA component [EC:2.7.1.191]
Q5A_001825	-0.784054272	0.000128357	0.01343315	cysQ; 3'(2'),5'-bisphosphate nucleotidase [EC:3.1.3.7]
Q5A_010700	-0.774422547	0.000383897	0.03209508	htpX; heat shock protein HtpX [EC:3.4.24.-]
Q5A_017530	-0.772978268	0.000651408	0.04732198	yfbT; sugar-phosphatase [EC:3.1.3.23]
Q5A_014745	-0.765542298	0.000232489	0.02134478	PTS-Man-EIID; PTS system, mannose-specific IID component
Q5A_014680	-0.744259112	0.000436668	0.03467316	uncharacterized protein
Q5A_015210	0.739892297	0.000435304	0.03467316	efeO; iron uptake system component EfeO
Q5A_007955	0.764324308	0.000306094	0.02663193	manB; phosphomannomutase [EC:5.4.2.8]
Q5A_016985	0.76993376	0.000180387	0.0175292	sbcB; exodeoxyribonuclease I [EC:3.1.11.1]
Q5A_019145	0.806510985	0.000131278	0.01364283	pepB; PepB aminopeptidase [EC:3.4.11.23]
Q5A_024815	0.823927599	0.000110024	0.01193125	argG; argininosuccinate synthase [EC:6.3.4.5]
Q5A_025450	0.865412879	0.000411826	0.0333134	gidB; 16S rRNA (guanine527-N7)-methyltransferase [EC:2.1.1.170]
Q5A_013920	0.874038701	8.20E-05	0.00952465	rluB; 23S rRNA pseudouridine2605 synthase [EC:5.4.99.22]
Q5A_000015	0.882396507	0.000271645	0.02457605	recF; DNA replication and repair protein RecF
Q5A_019090	0.885515526	4.51E-05	0.00603012	gcpE; (E)-4-hydroxy-3-methylbut-2-enyl-diphosphate synthase [EC:1.17.7.1 1.17.7.3]
Q5A_022525	0.919216585	0.000150986	0.01516709	exuT; MFS transporter, ACS family, hexuronate transporter
Q5A_025475	0.919870586	0.000183702	0.01773611	ATPF1D; F-type H+-transporting ATPase subunit delta
Q5A_023555	0.938796986	0.000428137	0.03444664	RP-S4; small subunit ribosomal protein S4
Q5A_023550	0.947685318	0.000356219	0.03011761	rpoA; DNA-directed RNA polymerase subunit alpha [EC:2.7.7.6]
Q5A_007960	0.956964677	0.000620997	0.04568884	ABC-2.LPSE.P; lipopolysaccharide transport system permease protein
Q5A_022100	0.958249224	0.000123936	0.01324783	exbD; biopolymer transport protein ExbD
Q5A_005985	0.961851385	0.000159021	0.01578623	rlpA; rare lipoprotein A
Q5A_003435	0.965989248	4.79E-05	0.00623402	degP; serine protease Do [EC:3.4.21.107]
Q5A_023610	0.996820178	0.000401521	0.03283475	RP-L24; large subunit ribosomal protein L24
Q5A_003275	1.011595864	0.000138069	0.01424971	murE; UDP-N-acetylmuramoyl-L-alanyl-D-glutamate--2,6-diaminopimelate ligase [EC:6.3.2.13]
Q5A_020635	1.02163108	0.000394468	0.03245928	KARS; lysyl-tRNA synthetase, class II [EC:6.1.1.6]
Q5A_003765	1.031375231	0.000593738	0.0439866	RP-S16; small subunit ribosomal protein S16
Q5A_000705	1.032100444	0.000508445	0.0390199	E2.7.7.24; glucose-1-phosphate thymidylyltransferase [EC:2.7.7.24]
Q5A_001315	1.073484442	3.28E-05	0.00462965	rpoB; DNA-directed RNA polymerase subunit beta [EC:2.7.7.6]
Q5A_004935	1.076320393	0.000216005	0.02040276	cyoC; cytochrome o ubiquinol oxidase subunit III
Q5A_004580	1.086598904	0.000114091	0.01228325	uncharacterized protein
Q5A_019925	1.098903785	7.38E-05	0.00890562	dnaE; DNA polymerase III subunit alpha [EC:2.7.7.7]
Q5A_010715	1.104205831	5.73E-05	0.00715045	uncharacterized protein
Q5A_022645	1.109120361	2.59E-05	0.00372764	gltD; glutamate synthase (NADPH/NADH) small chain [EC:1.4.1.13 1.4.1.14]
Q5A_005990	1.11082645	3.44E-05	0.00481475	rodA; rod shape determining protein RodA
Q5A_025480	1.114497584	3.78E-05	0.00519142	ATPF1A; F-type H+-transporting ATPase subunit alpha [EC:3.6.3.14]
Q5A_025495	1.115437397	9.85E-06	0.00165079	ATPF1E; F-type H+-transporting ATPase subunit epsilon
Q5A_010095	1.123318289	0.000468845	0.0367344	phoQ; two-component system, OmpR family, sensor histidine kinase PhoQ [EC:2.7.13.3]
Q5A_020770	1.124098577	8.92E-05	0.00993022	gcvH; glycine cleavage system H protein
Q5A_020225	1.126066465	5.22E-05	0.00667858	bisC; biotin/methionine sulfoxide reductase [EC:1.-.-.-]
Q5A_000715	1.14106216	6.48E-05	0.00794402	wecE; dTDP-4-amino-4,6-dideoxygalactose transaminase [EC:2.6.1.59]

Gene	logFC	PValue	FDR	Function
Q5A_007965	1.159588453	7.48E-07	0.00018055	ABC-2.LPSE.A; lipopolysaccharide transport system ATP-binding protein
Q5A_018270	1.163190653	0.00067399	0.04825964	cysM; cysteine synthase B [EC:2.5.1.47]
Q5A_025490	1.181219213	6.49E-06	0.00115707	ATPF1B; F-type H+-transporting ATPase subunit beta [EC:3.6.3.14]
Q5A_023600	1.189779741	0.000308508	0.02668686	RP-S14; small subunit ribosomal protein S14
Q5A_025485	1.231988595	2.69E-06	0.0005585	ATPF1G; F-type H+-transporting ATPase subunit gamma
Q5A_021225	1.249899624	2.79E-06	0.00057138	DLAT; pyruvate dehydrogenase E2 component (dihydrolipoamide acetyltransferase) [EC:2.3.1.12]
Q5A_007970	1.253396764	9.88E-06	0.00165079	wbdD; O-antigen chain-terminating methyltransferase [EC:2.1.1.- 2.1.1.294 2.7.1.181]
Q5A_023595	1.255512256	0.000151012	0.01516709	RP-S8; small subunit ribosomal protein S8
Q5A_003510	1.269529949	8.75E-05	0.00991439	ENO; enolase [EC:4.2.1.11]
Q5A_023665	1.279074138	0.000580067	0.04357256	RP-L3; large subunit ribosomal protein L3
Q5A_007565	1.311440579	0.000486067	0.03768909	betB; betaine-aldehyde dehydrogenase [EC:1.2.1.8]
Q5A_010135	1.344256844	4.57E-05	0.00605029	purB; adenylosuccinate lyase [EC:4.3.2.2]
Q5A_023590	1.394642505	6.27E-06	0.00114494	RP-L6; large subunit ribosomal protein L6
Q5A_013425	1.402843453	8.81E-05	0.00991439	livK; branched-chain amino acid transport system substrate-binding protein
Q5A_013955	1.41352829	4.21E-05	0.0057213	trpD; anthranilate phosphoribosyltransferase [EC:2.4.2.18]
Q5A_023150	1.413695908	0.000216775	0.02040276	N/A
Q5A_005595	1.429913055	0.000502136	0.03873437	N/A
Q5A_025240	1.448793539	0.000386643	0.03214509	trmH; tRNA (guanosine-2-O-)-methyltransferase [EC:2.1.1.34]
Q5A_023585	1.450431734	5.46E-07	0.00013406	RP-L18; large subunit ribosomal protein L18
Q5A_013960	1.483167811	2.70E-09	1.01E-06	trpCF; indole-3-glycerol phosphate synthase
Q5A_002020	1.489299833	7.87E-05	0.00927786	truB; tRNA pseudouridine55 synthase [EC:5.4.99.25]
Q5A_001160	1.491198888	7.36E-10	3.15E-07	rfaH; transcriptional antiterminator RfaH
Q5A_016980	1.494395971	0.000333217	0.02833289	holE; DNA polymerase III subunit theta [EC:2.7.7.7]
Q5A_023575	1.520736432	8.56E-07	0.00020334	RP-L15; large subunit ribosomal protein L15
Q5A_023430	1.535747999	0.000323099	0.02778839	aceB; malate synthase [EC:2.3.3.9]
Q5A_023660	1.545475067	0.00015085	0.01516709	RP-L4; large subunit ribosomal protein L4
Q5A_023580	1.598435296	1.08E-06	0.000246	RP-S5; small subunit ribosomal protein S5
Q5A_018150	1.616349566	8.70E-05	0.00991439	fes; enterochelin esterase and related enzymes
Q5A_010010	1.633157137	2.07E-10	1.07E-07	kdsA; 2-dehydro-3-deoxyphosphooctonate aldolase (KDO 8-P synthase) [EC:2.5.1.55]
Q5A_010365	1.661337332	0.000437904	0.03467316	cspA; cold shock protein (beta-ribbon, CspA family)
Q5A_025245	1.673046601	0.000140408	0.01439184	recG; ATP-dependent DNA helicase RecG [EC:3.6.4.12]
Q5A_023655	1.706448858	2.80E-05	0.00399534	RP-L23; large subunit ribosomal protein L23
Q5A_024965	1.722227382	9.71E-10	3.93E-07	cysP; sulfate transport system substrate-binding protein
Q5A_008630	1.768955327	0.000585238	0.04357256	ssuD; alkanesulfonate monooxygenase [EC:1.14.14.5]
Q5A_023630	1.798199582	2.19E-07	5.75E-05	RP-L16; large subunit ribosomal protein L16
Q5A_023650	1.81282525	8.88E-07	0.00020768	RP-L2; large subunit ribosomal protein L2
Q5A_007015	1.887269012	1.19E-05	0.00189254	metQ; D-methionine transport system substrate-binding protein
Q5A_019155	1.923841527	5.19E-06	0.0009836	fdx; ferredoxin, 2Fe-2S
Q5A_013965	1.936374402	4.44E-13	3.16E-10	trpB; tryptophan synthase beta chain [EC:4.2.1.20]
Q5A_012540	1.952549176	8.72E-11	4.83E-08	aldB; aldehyde dehydrogenase [EC:1.2.1.-]
Q5A_023625	1.955694281	3.46E-11	2.07E-08	RP-L29; large subunit ribosomal protein L29
Q5A_012535	1.958198587	1.22E-09	4.82E-07	adhP; alcohol dehydrogenase, propanol-preferring [EC:1.1.1.1]
Q5A_025575	1.972560444	8.98E-05	0.00993022	mnmE; tRNA modification GTPase [EC:3.6.-.-]
Q5A_018170	1.998960436	9.93E-06	0.00165079	alsD; acetolactate decarboxylase [EC:4.1.1.5]
Q5A_023635	2.001100364	9.61E-10	3.93E-07	RP-S3; small subunit ribosomal protein S3
Q5A_013970	2.005143785	4.59E-07	0.00011445	trpA; tryptophan synthase alpha chain [EC:4.2.1.20]
Q5A_002015	2.008984415	1.07E-10	5.71E-08	rbfA; ribosome-binding factor A
Q5A_023645	2.073454609	5.03E-09	1.84E-06	RP-S19; small subunit ribosomal protein S19
Q5A_018290	2.078549829	9.21E-05	0.01005642	cysP; sulfate transport system substrate-binding protein
Q5A_019160	2.097698613	3.77E-08	1.23E-05	hscA; molecular chaperone HscA
Q5A_023640	2.114113863	4.69E-10	2.19E-07	RP-L22; large subunit ribosomal protein L22
Q5A_023620	2.11451368	7.20E-08	2.16E-05	RP-S17; small subunit ribosomal protein S17
Q5A_013945	2.171591652	2.32E-15	2.48E-12	trpE; anthranilate synthase component I [EC:4.1.3.27]
Q5A_007560	2.223927239	1.05E-06	0.0002421	betI; TetR/AcrR family transcriptional regulator, transcriptional repressor of bet genes
Q5A_023420	2.26789094	4.83E-06	0.00093786	aceK; isocitrate dehydrogenase kinase/phosphatase [EC:2.7.11.5 3.1.3.-]
Q5A_023425	2.27591751	6.98E-10	3.07E-07	E4.1.3.1; isocitrate lyase [EC:4.1.3.1]
Q5A_008095	2.28160194	3.26E-11	2.03E-08	CTH; cystathionine gamma-lyase [EC:4.4.1.1]
Q5A_012240	2.29361659	2.90E-13	2.41E-10	ABC.SS.S; simple sugar transport system substrate-binding protein
Q5A_007025	2.337387855	4.36E-05	0.00587802	metN; D-methionine transport system ATP-binding protein
Q5A_006275	2.343443956	2.85E-07	7.22E-05	kdpA; K+-transporting ATPase ATPase A chain [EC:3.6.3.12]
Q5A_025535	2.370161716	6.18E-06	0.00114135	ABC.PA.S; polar amino acid transport system substrate-binding protein
Q5A_003565	2.383715546	1.80E-15	2.07E-12	cysJ; sulfite reductase (NADPH) flavoprotein alpha-component [EC:1.8.1.2]
Q5A_008635	2.416532155	0.000159286	0.01578623	ssuA; sulfonate transport system substrate-binding protein
Q5A_009885	2.468386668	1.27E-24	2.71E-21	metQ; D-methionine transport system substrate-binding protein
Q5A_013150	2.488430101	0.000272611	0.02457605	rbsB; ribose transport system substrate-binding protein
Q5A_005920	2.609600567	3.92E-12	2.67E-09	yxeP; uncharacterized hydrolase [EC:3.-.-.-]
Q5A_003600	2.638693549	2.27E-09	8.72E-07	cysC; adenylylsulfate kinase [EC:2.7.1.25]
Q5A_013950	2.684995402	3.42E-08	1.14E-05	trpG; anthranilate synthase component II [EC:4.1.3.27]
Q5A_003585	2.693693766	2.94E-15	2.93E-12	cysG; uroporphyrin-III C-methyltransferase
Q5A_008100	2.795399648	5.94E-21	8.08E-18	CBS; cystathionine beta-synthase [EC:4.2.1.22]
Q5A_015155	2.962650632	6.70E-11	3.85E-08	N/A
Q5A_018285	3.206409719	2.81E-16	3.50E-13	cysU; sulfate transport system permease protein
Q5A_003570	3.27941167	4.09E-25	1.02E-21	cysI; sulfite reductase (NADPH) hemoprotein beta-component [EC:1.8.1.2]
Q5A_023765	3.3435085	2.48E-07	6.41E-05	tauC; taurine transport system permease protein
Q5A_003575	3.414713777	1.20E-21	1.80E-18	cysH; phosphoadenosine phosphosulfate reductase [EC:1.8.4.8 1.8.4.10]
Q5A_013415	3.52258229	2.39E-05	0.00346779	livG; branched-chain amino acid transport system ATP-binding protein
Q5A_003590	3.579275381	4.44E-27	1.33E-23	cysD; sulfate adenylyltransferase subunit 2 [EC:2.7.7.4]
Q5A_012175	3.63206883	1.78E-05	0.00265913	ABC.PA.A; polar amino acid transport system ATP-binding protein [EC:3.6.3.21]
Q5A_013405	3.660212984	2.24E-13	1.98E-10	livH; branched-chain amino acid transport system permease protein
Q5A_018280	3.758245711	1.76E-23	3.29E-20	cysW; sulfate transport system permease protein
Q5A_003595	3.86355858	5.63E-28	2.10E-24	cysN; sulfate adenylyltransferase subunit 1 [EC:2.7.7.4]
Q5A_008640	3.97607918	1.13E-06	0.00024804	ssuE; FMN reductase [EC:1.5.1.38]
Q5A_013420	4.062233442	1.23E-07	3.40E-05	livF; branched-chain amino acid transport system ATP-binding protein
Q5A_012185	4.110365562	1.17E-07	3.30E-05	ABC.PA.S; polar amino acid transport system substrate-binding protein
Q5A_023775	4.137817367	3.48E-33	1.74E-29	tauA; taurine transport system substrate-binding protein
Q5A_015160	4.227876863	1.43E-39	2.14E-35	cbl; LysR family transcriptional regulator, cys regulon transcriptional activator
Q5A_018275	4.32009885	7.32E-37	5.48E-33	cysA; sulfate transport system ATP-binding protein [EC:3.6.3.25]
Q5A_012950	4.563975017	2.44E-10	1.18E-07	cysE; serine O-acetyltransferase [EC:2.3.1.30]
Q5A_012180	4.668051343	1.31E-06	0.00028492	ABC.PA.P; polar amino acid transport system permease protein
Q5A_013410	4.749568629	4.29E-08	1.36E-05	livM; branched-chain amino acid transport system permease protein
Q5A_023760	5.264884391	9.51E-22	1.58E-18	tauD; taurine dioxygenase [EC:1.14.11.17]
Q5A_023770	7.56664463	1.36E-13	1.27E-10	tauB; taurine transport system ATP-binding protein [EC:3.6.3.36]
