# Supplementary material for: Exploring the Interspecific Interactions and the Metabolome of the Soil Isolate Hylemonella gracilis
Source: mSystems. 2022 Dec 20;8(1):e00574-22. doi: 10.1128/msystems.00574-22 (PMC9948732; doi:10.1128/msystems.00574-22)
Supplement: TABLE S4 [file msystems.00574-22-s0007.pdf]

Supplementary Table 4: Significantly differentially expressed genes of *H. gracilis* responding to *Serratia plymuthica* PRI-2C at day 5.

Gene	logFC	PValue	FDR	Function
hylg_3594	-10.30347602	1.79E-09	2.97E-06	xylB; D-xylose 1-dehydrogenase [EC:1.1.1.175]
hylg_1896	-9.05270584	3.82E-06	0.00173071	livF; branched-chain amino acid transport system ATP-binding protein
hylg_681	-8.967993981	1.91E-05	0.00681266	rlmN; 23S rRNA (adenine2503-C2)-methyltransferase [EC:2.1.1.192]
hylg_2585	-8.951345037	2.01E-05	0.00700548	rbsC; ribose transport system permease protein
hylg_888	-8.916187339	7.17E-06	0.00275048	flhA; flagellar biosynthesis protein FlhA
hylg_2519	-8.822014508	0.000219866	0.04112861	ybeB; ribosome-associated protein
hylg_2782	-8.738718593	8.00E-05	0.02064782	uncharacterized protein
hylg_1702	-8.701055125	0.000167787	0.03303859	hisH; glutamine amidotransferase [EC:2.4.2.-]
hylg_3268	-8.665105299	4.29E-05	0.01337529	glnB; nitrogen regulatory protein P-II 1
hylg_76	-8.529781325	0.000125752	0.02727353	NTE family protein
hylg_1992	-8.287022835	0.000271079	0.0462024	UGDH; UDPglucose 6-dehydrogenase [EC:1.1.1.22]
hylg_2781	-4.886910881	1.08E-09	2.31E-06	cheY; two-component system, chemotaxis family, chemotaxis protein CheY
hylg_3601	-3.960678302	9.61E-05	0.02358015	yiaN; TRAP-type transport system large permease protein
hylg_3527	-3.848207509	0.000108449	0.02496838	argG; argininosuccinate synthase [EC:6.3.4.5]
hylg_2468	-3.624361126	6.29E-06	0.00254507	btuB; vitamin B12 transporter
hylg_1855	-3.563911838	0.00011898	0.02618433	VARS; valyl-tRNA synthetase [EC:6.1.1.9]
hylg_3497	-3.541208019	0.000152069	0.03117422	N/A
hylg_54	-3.530398733	0.000256743	0.04467628	benE; benzoate membrane transport protein
hylg_1780	-3.441042352	0.00013748	0.02939115	polA; DNA polymerase I [EC:2.7.7.7]
hylg_2929	-3.393004036	6.98E-05	0.01900203	smc; chromosome segregation protein
hylg_2586	-3.377821679	7.56E-05	0.02006357	rbsA; ribose transport system ATP-binding protein [EC:3.6.3.17]
hylg_2197	-3.328784638	2.33E-06	0.00108951	hlpA; outer membrane protein
hylg_535	-3.275554622	6.08E-05	0.01750013	livK; branched-chain amino acid transport system substrate-binding protein
hylg_1719	-3.254220622	4.84E-06	0.00201119	sspA; stringent starvation protein A
hylg_538	-2.949406851	0.000204119	0.03866636	glnA; glutamine synthetase [EC:6.3.1.2]
hylg_3080	-2.916611874	0.00024938	0.04393638	sdhC; succinate dehydrogenase / fumarate reductase, cytochrome b subunit
hylg_1625	-2.90876304	2.39E-05	0.00813567	metK; S-adenosylmethionine synthetase [EC:2.5.1.6]
hylg_1958	-2.845623805	8.52E-05	0.0216103	fimV; pilus assembly protein FimV
hylg_2584	-2.8004223	0.000291159	0.04788124	rbsB; ribose transport system substrate-binding protein
hylg_3274	-2.715164625	0.000162179	0.03236019	ppa; inorganic pyrophosphatase [EC:3.6.1.1]
hylg_1357	-2.646079415	0.000249555	0.04393638	N/A
hylg_1747	-2.24362962	0.000145461	0.03023366	N/A
hylg_1116	-2.149397607	0.000230074	0.04250696	E3.3.1.1; adenosylhomocysteinase [EC:3.3.1.1]
hylg_3616	-2.133709269	0.000238398	0.04315128	N/A
hylg_197	-2.014259372	6.94E-06	0.00273302	coxB; cytochrome c oxidase subunit II [EC:1.9.3.1]
hylg_3051	-1.961997657	3.78E-05	0.01229204	ENO; enolase [EC:4.2.1.11]
hylg_2411	1.836427686	0.000271688	0.0462024	iscS; cysteine desulfurase [EC:2.8.1.7]
hylg_2413	2.142314215	0.000103623	0.02461468	iscA; iron-sulfur cluster assembly protein
hylg_956	2.252713163	6.32E-05	0.01784859	N/A
hylg_3349	2.513218505	1.03E-05	0.00387033	RP-S21; small subunit ribosomal protein S21
hylg_244	2.645172803	1.82E-06	0.00090883	rluF; 23S rRNA pseudouridine2604 synthase [EC:5.4.99.21]
hylg_3064	2.836010247	4.50E-05	0.01374931	N/A
hylg_764	2.845097872	1.69E-06	0.00086973	ksgA; 16S rRNA (adenine1518-N6/adenine1519-N6)-dimethyltransferase [EC:2.1.1.182]
hylg_2414	2.990210312	9.27E-05	0.02312815	hscB; molecular chaperone HscB
hylg_3350	3.165994836	2.98E-07	0.00017819	uncharacterized protein
hylg_3472	3.309925768	7.35E-07	0.00042277	bpeF; multidrug efflux pump
hylg_2415	3.400742126	1.22E-06	0.0006771	hscA; molecular chaperone HscA
hylg_3352	3.581267445	4.51E-08	4.22E-05	gudD; glucarate dehydratase [EC:4.2.1.40]
hylg_359	3.815734971	1.02E-07	7.36E-05	cheW; purine-binding chemotaxis protein CheW
hylg_3471	3.830015991	3.18E-05	0.01057743	bpeE; membrane fusion protein, multidrug efflux system
hylg_3068	3.969695582	4.42E-06	0.00189132	trmD; tRNA (guanine37-N1)-methyltransferase [EC:2.1.1.228]
hylg_1092	5.118307349	1.69E-09	2.97E-06	N/A
hylg_360	5.190346574	5.26E-05	0.01543046	mcp; methyl-accepting chemotaxis protein
hylg_1563	5.297224632	1.03E-07	7.36E-05	mcp; methyl-accepting chemotaxis protein
hylg_3477	5.333738341	1.97E-06	0.00094974	acrA; membrane fusion protein, multidrug efflux system
hylg_2376	5.556284733	2.87E-09	4.29E-06	N/A
hylg_357	5.920231993	7.62E-11	2.28E-07	N/A
hylg_363	5.987661764	1.51E-12	7.53E-09	mcp; methyl-accepting chemotaxis protein
hylg_1561	6.333104946	4.06E-10	1.01E-06	N/A
hylg_358	6.400072284	3.67E-13	2.75E-09	N/A
hylg_361	7.03578819	7.95E-09	1.02E-05	cheW; purine-binding chemotaxis protein CheW
