# Supplementary material for: Exploring the Interspecific Interactions and the Metabolome of the Soil Isolate Hylemonella gracilis
Source: mSystems. 2022 Dec 20;8(1):e00574-22. doi: 10.1128/msystems.00574-22 (PMC9948732; doi:10.1128/msystems.00574-22)
Supplement: TABLE S5 [file msystems.00574-22-s0008.pdf]

Supplementary Table 5: Significantly up- or down regulated genes of *H. gracilis* responding to *Serratia plymuthica* PRI-2C at day 10.

Gene	logFC	PValue	FDR	Function
hylg_1092	4.028021412	5.89E-07	0.00152989	N/A
hylg_1561	4.825700429	3.64E-06	0.00680041	N/A
hylg_1562	4.569714447	8.80E-07	0.00188109	cheW; purine-binding chemotaxis protein CheW
hylg_1563	5.426932759	7.79E-08	0.00029152	mcp; methyl-accepting chemotaxis protein
hylg_2376	3.649019855	1.95E-05	0.02289822	N/A
hylg_357	4.707340509	6.13E-07	0.00152989	N/A
hylg_358	5.714802483	2.38E-09	1.78E-05	N/A
hylg_359	3.924767967	9.74E-06	0.01458213	cheW; purine-binding chemotaxis protein CheW
hylg_361	6.080192446	1.41E-05	0.01917412	cheW; purine-binding chemotaxis protein CheW
hylg_363	5.259687546	4.19E-10	6.27E-06	mcp; methyl-accepting chemotaxis protein
