# Supplementary material for: Exploring the Interspecific Interactions and the Metabolome of the Soil Isolate Hylemonella gracilis
Source: mSystems. 2022 Dec 20;8(1):e00574-22. doi: 10.1128/msystems.00574-22 (PMC9948732; doi:10.1128/msystems.00574-22)
Supplement: FIG S2 [file msystems.00574-22-s0003.pdf]

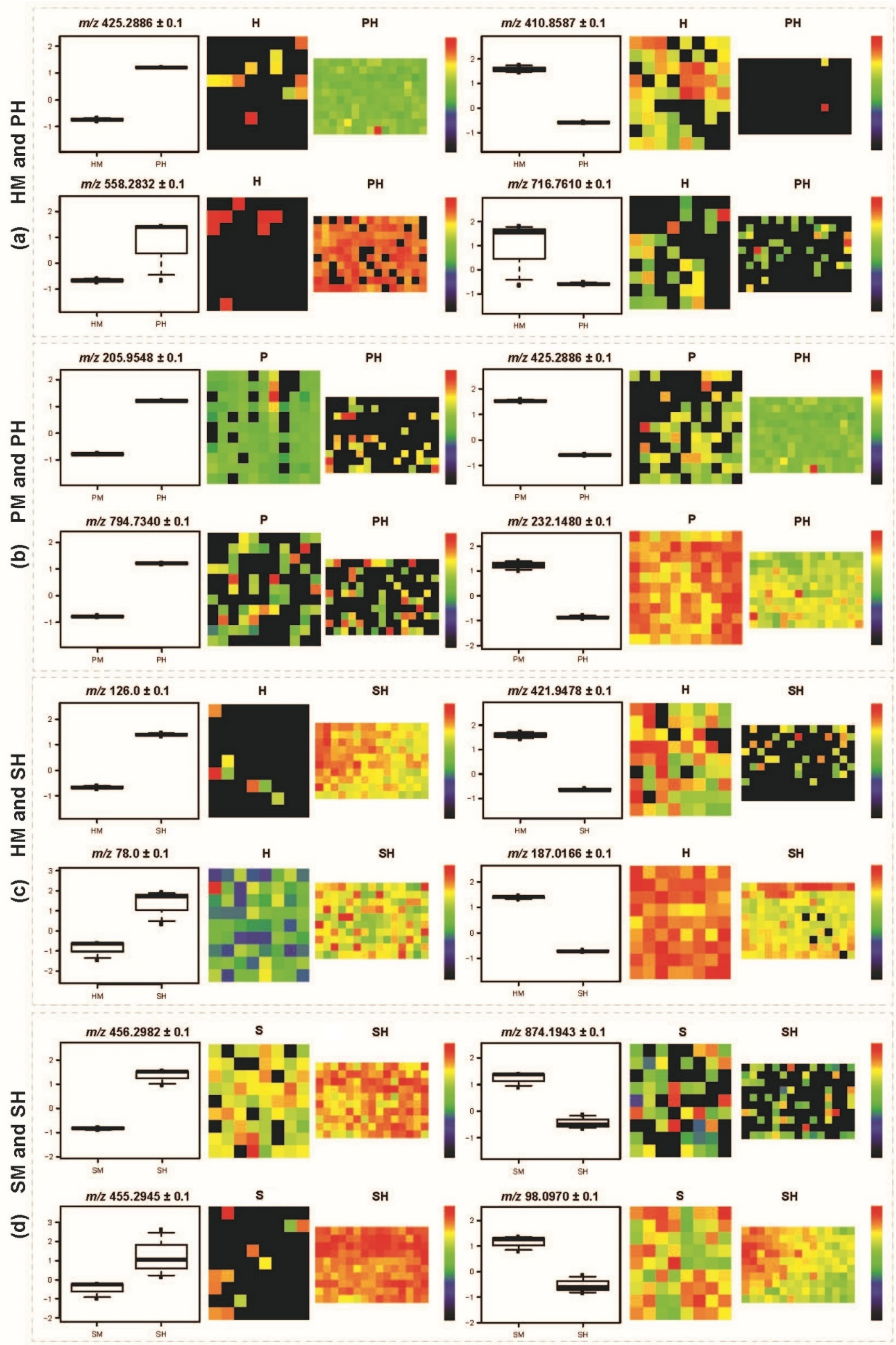

Supplementary Figure 2: Box plots for the significantly differentially abundant metabolites and their corresponding ion intensity maps found using Mass spectrometry imaging (MSI) during the interaction of *H. gracilis* with *Paenibacillus* sp. AD87 and *S. plymuthica* PRI-2C.
