# Supplementary material for: Exploring the Interspecific Interactions and the Metabolome of the Soil Isolate Hylemonella gracilis
Source: mSystems. 2022 Dec 20;8(1):e00574-22. doi: 10.1128/msystems.00574-22 (PMC9948732; doi:10.1128/msystems.00574-22)
Supplement: FIG S1 [file msystems.00574-22-s0002.pdf]

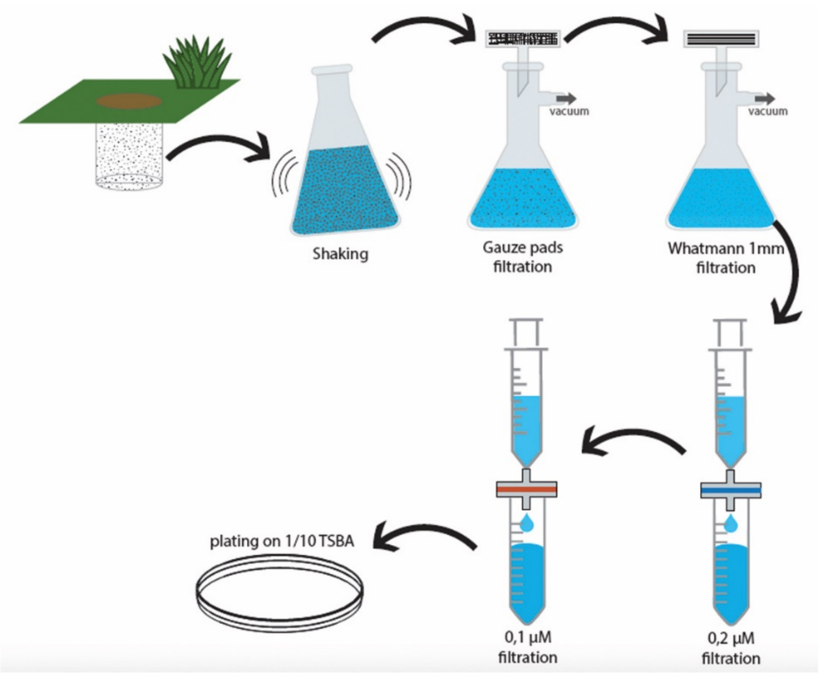

Supplementary Figure 1: Schematic overview of the applied isolation method used to isolate *H. gracilis* from soil.
