# Supplementary material for: Exploring the Interspecific Interactions and the Metabolome of the Soil Isolate Hylemonella gracilis
Source: mSystems. 2022 Dec 20;8(1):e00574-22. doi: 10.1128/msystems.00574-22 (PMC9948732; doi:10.1128/msystems.00574-22)
Supplement: TABLE S6 [file msystems.00574-22-s0009.pdf]

Supplementary Table 6: Significantly up- or down regulated genes of *H. gracilis* responding to *Paenibacillus* sp. AD87 at day 5.

Gene	logFC	PValue	FDR	Function
hylg_283	1.83868209	1.21E-05	0.02840838	soxY; sulfur-oxidizing protein SoxY
hylg_2715	3.18297333	3.22E-08	0.000482259	pobA; p-hydroxybenzoate 3-monooxygenase [EC:1.14.13.2]
hylg_1092	3.66916806	3.70E-06	0.013834781	
hylg_1561	3.88090845	2.32E-05	0.043402807	
hylg_689	3.90578955	5.43E-07	0.002708319	
hylg_1563	3.91597386	2.64E-05	0.043946331	mcp; methyl-accepting chemotaxis protein
hylg_361	4.72545337	1.33E-05	0.02840838	cheW; purine-binding chemotaxis protein CheW
hylg_2376	4.8197097	9.29E-08	0.000694792	
