# Supplementary material for: Exploring the Interspecific Interactions and the Metabolome of the Soil Isolate Hylemonella gracilis
Source: mSystems. 2022 Dec 20;8(1):e00574-22. doi: 10.1128/msystems.00574-22 (PMC9948732; doi:10.1128/msystems.00574-22)
Supplement: TEXT S1 [file msystems.00574-22-s0001.docx]

**Supplementary Methods**

**Pathway annotations**

For pathway annotations, BlastKOALA was used (<https://www.kegg.jp/blastkoala/>) (1). FASTA files of the Protein sequences of the significantly differentially expressed genes were extracted from GenBank (<https://www.ncbi.nlm.nih.gov/genbank/>) (2) and merged into a new FASTA file corresponding to each comparison group. The FASTA files were uploaded to BlastKOALA and a search was performed against the prokaryotes KEGG GENES database file.

**GC-Q-TOF data analysis**

Volatile organic compounds (VOCs) were trapped by using glass Petri dishes (3). At the top of the Petri dishes the lid was connected with an outlet to a steel trap containing 150 mg Tenax TA and 150 mg Carbopack B (Markes International Ltd). The Tenax steel traps were added after four and nine days of incubation and collected at day five and ten of incubation. As controls glass Petri dishes containing 1/10^th^ TSBA without bacteria were used. The VOCs were desorbed from the Tenax using an automated thermos desorption unit (Unity TD-100, Markes International Ltd., Llantrisant, UK). The desorbed volatiles were introduced into the GC-QTOF (model Agilent 7890B GC and the Agilent 7200A QTOF, Santa Clara, USA) and measured as described in (4). For VOC data analysis and compound identification Mass-spectra were extracted with MassHunter Qualitative Analysis Software V B.06.00 Build 6.0.633.0 (Agilent Technologies, Santa Clara, USA) and exported as netCDF files using the MassHunter GC/MS (GC-AIA) Translator B.07.00 SP2 163.. The netCDF files were imported to MZmine V2.24 (Copyright © 2005-2015 MZmine Development Team) (5) and compounds were identified based on their mass spectra and by their linear retention indexes (LRI) in combination with two mass-spectral-libraries: NIST 2014 V2.20 (National Institute of Standards and Technology, USA [http://www.nist.gov](http://www.nist.gov/)). The LRI values were calculated using AMDIS 2.72 (National Institute of Standards and Technology, USA). Peak lists containing the mass features of each treatment were exported in csv file format and uploaded to Metaboanalyst V3.0 ([www.metaboanalyst.ca](http://www.metaboanalyst.ca)) running on a local UNIX server for statistical analysis (6). To identify significantly differentially (OK?) abundant masses ONE-WAY-ANOVA with post-hoc TUKEY test was performed between the data sets.

**Direct Analysis in Real Time Mass Spectrometry (DART-MS) data analysis**

DART-MS data analysis was performed with Xcalibur 2.2 (Thermo) and files were exported as mzdata files and further analyzed using MZmine V2.24 (Copyright © 2005-2015 MZmine Development Team) (5). Compounds were identified via their mass spectra using the online KEGG database (7, 8). After deconvolution and mass identification, peak lists containing the mass features of each treatment were exported in csv file format and uploaded to a local copy of Metaboanalyst V3.0 ([www.metaboanalyst.ca](http://www.metaboanalyst.ca)) running on a local UNIX server for statistical analysis (6). To identify significant abundant masses ONE-WAY-ANOVA with post-hoc TUKEY test was performed between the data sets. Masses were considered to be statistical relevant if p- and FDR- values were ≤ 0.05.

**References Supplementary Material**

1. Kanehisa M, Sato Y, Morishima K. 2016. BlastKOALA and GhostKOALA: KEGG Tools for Functional Characterization of Genome and Metagenome Sequences. J Mol Biol 428:726-731.

2. Sayers EW, Cavanaugh M, Clark K, Ostell J, Pruitt KD, Karsch-Mizrachi I. 2018. GenBank. Nucleic Acids Research 47:D94-D99.

3. Garbeva P, Hordijk C, Gerards S, de Boer W. 2014. Volatiles produced by the mycophagous soil bacterium Collimonas. Fems Microbiology Ecology 87.

4. Tyc O, Zweers H, De Boer W, Garbeva P. 2015. Volatiles in inter-specific bacterial interactions. Frontiers in Microbiology 6.

5. Pluskal T, Castillo S, Villar-Briones A, Oresic M. 2010. MZmine 2: modular framework for processing, visualizing, and analyzing mass spectrometry-based molecular profile data. Bmc Bioinformatics 11:395.

6. Xia J, Sinelnikov IV, Han B, Wishart DS. 2015. MetaboAnalyst 3.0-making metabolomics more meaningful. Nucleic Acids Research 43:W251-7.

7. Anonymous. The KEGG Database, ‘In Silico’ Simulation of Biological Processes doi:10.1002/0470857897.ch8.

8. Kanehisa M. 2008. The KEGG Database. *In* Novartis Foundation GBaJAG (ed), The KEGG Database In ‘In Silico’ Simulation of Biological Processes doi:10.1002/0470857897.ch8.
